# Osteocyte-specific gasdermin D deletion accelerates osteoarthritis via promoting subchondral inflammation and remodeling

**DOI:** 10.1038/s41413-026-00566-w

**Published:** 2026-07-28

**Authors:** Yuki Ogawa, Taku Ebata, Taiki Tokuhiro, Liyile Chen, Ryota Suzuki, Yuki Fujie, Masaya Nakajo, Tsutomu Endo, Hend Alhasan, Masanari Hamasaki, Daisuke Takahashi, Koji Iwasaki, Ken Kadoya, Tomohiro Onodera, Norimasa Iwasaki, M. Alaa Terkawi

**Affiliations:** Department of Orthopaedic Surgery, Faculty of Medicine and Graduate School of Medicine, Hokkaido University, Sapporo, Japan

**Keywords:** Pathogenesis, Metabolic bone disease

## Abstract

Subchondral bone remodeling in the early stages of osteoarthritis (OA) is characterized by elevated bone turnover and is closely associated with osteocyte function within the subchondral bone. However, beyond the canonical role of gasdermin D (GSDMD) in mediating pyroptosis in immune cells, its function in osteocytes remains poorly understood. Accordingly, this study aimed to determine whether osteocyte-derived GSDMD regulates subchondral bone homeostasis or contributes to pathological remodeling during osteoarthritis progression. GSDMD expression was evident in osteocytes within the subchondral bone in both clinical and experimental models of OA, as well as in osteocytes co-cultured with stimulated chondrocytes, suggesting a potential role for osteocyte-derived GSDMD in the bone microenvironment. Remarkably, specific deletion of *Gsdmd* in osteocytes led to increased bone remodeling, which was accompanied by cartilage degeneration in an OA model, as well as bone loss in an osteoporosis model. Mechanistically, *Gsdmd*-deficient osteocytes displayed enhanced activation of CARD9/NF-κB signaling under inflammatory stimulation, leading to increased production of inflammatory mediators and osteoclastogenic factors that disrupted osteoblast-osteoclast coupling. These changes induced high-turnover subchondral bone remodeling, altered joint mechanical properties, and accelerated cartilage degeneration. Collectively, our findings identify osteocyte-derived GSDMD as an important regulator of subchondral bone remodeling and suggest that loss of this regulatory mechanism exacerbates OA progression. These results further indicate that therapeutic strategies targeting GSDMD should be approached with caution due to potential effects on bone remodeling and inflammatory signaling.

## Introduction

Osteoarthritis (OA) is a degenerative joint disease characterized by pain and stiffness, which can significantly impair daily activities and lead to disability. This disease is highly prevalent, affecting 7% of the global population, particularly individuals over the age of 55.^1^ Consequently, OA imposes a significant burden on society, encompassing healthcare expenditures and the economic impact of reduced productivity of society due to early retirement from work. While there are no pharmacological agents that can reverse cartilage damage, current therapeutic interventions aim to alleviate pain and enhance functionality and quality of life.^2^ Developing disease-modifying and specific treatments for OA remains a challenge, primarily due to our incomplete understanding of the cellular and molecular mechanisms that maintain and restore joint homeostasis.^3^

In addition to cartilage degeneration, the pathogenesis of OA includes dramatic changes in the microstructure of subchondral bone due to impairment of bone metabolism. These changes include increased subchondral bone sclerosis with thickening of the cortical plate and formation of osteophytes and bone cysts. Subchondral bone undergoes strictly balanced modeling and remodeling processes to maintain a healthy microstructure, which is important to provide mechanical and nutritional support for the cartilage and the joint. Therefore, alteration of the subchondral microenvironment due to impaired bone metabolism can exaggerate cartilage degeneration and disease progression. Subchondral bone lesions, including bone marrow edema and angiogenesis, appear to develop earlier than cartilage degeneration.^4,5^ In the early stage of OA, the remodeling process with an elevated turnover rate in the subchondral bone alters mechanical and biological signals in the joint, resulting in excessive cartilage degeneration.^5–7^ Thus, understanding the cellular and molecular changes in the subchondral bone during OA progression may provide valuable insights into disease pathogenesis and clues for the discovery of new therapeutic targets.

In mineralized bone tissues, including the subchondral bone, osteocytes are the most abundant cell type and serve as key regulators of bone homeostasis. They communicate with other cells using lacunar-canalicular systems to regulate and control mineralization in response to mechanical load.^8^ Gasdermin D (GSDMD), a member of the gasdermin family, has been extensively studied as the key executioner of pyroptosis, an inflammatory form of regulated cell death that plays fundamental roles in host defense and immune activation. Unlike apoptosis and necroptosis, pyroptosis is a lytic form of cell death triggered by pathogen-associated molecular patterns and damage-associated molecular patterns (DAMPs), which induce cell membrane rupture and the release of pro-inflammatory cytokines. In the context of OA, increased GSDMD expression and activation have been reported in joint-resident cells such as chondrocytes and synovial cells during OA progression, where GSDMD-mediated pyroptosis has been linked to joint inflammation and cartilage degeneration.^9^ However, growing evidence suggests that the biological functions of GSDMD are not limited to the execution of lytic cell death. Recent studies indicate that GSDMD can exert non-canonical regulatory roles, including the modulation of intracellular signaling pathways and the maintenance of cellular and tissue homeostasis in a context-dependent manner. These non-lytic functions appear to be particularly relevant in non-immune cells and may involve regulation of cytoskeletal dynamics, organelle homeostasis, and tissue integrity.^10,11^ These findings suggest that GSDMD exerts context-dependent physiological or pathological effects depending on the cellular environment and disease stage, rather than functioning as a uniformly pro-death factor. In this context, whether GSDMD plays a pathological role or instead serves a distinct homeostatic function in osteocytes-long-lived, terminally differentiated cells that orchestrate subchondral bone metabolism-remains largely unknown. In particular, the role of osteocyte-derived GSDMD in modulating subchondral bone homeostasis during OA progression has not been investigated. To address this knowledge gap, the present study examined the role of osteocyte-derived GSDMD in regulating and modulating subchondral bone homeostasis during OA progression.

## Results

### Induction of osteocyte pyroptosis in subchondral bone during OA progression

**Fig. 1:**
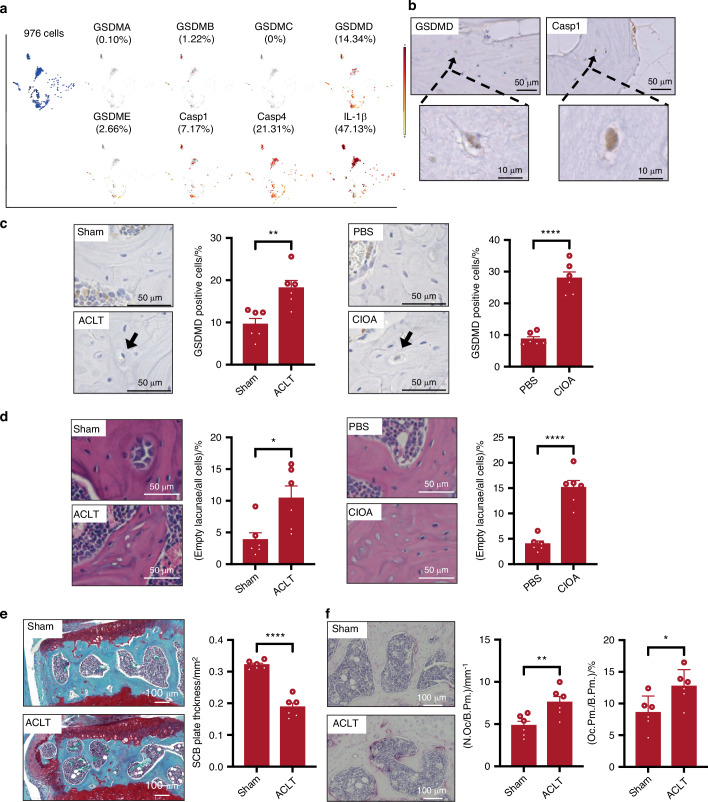
Detection of pyroptosis signaling-related molecules in osteocytes and histological changes in SCB during OA progression. **a** t-SNE plots of 976 osteocytes in the SCB of OA knees showing expression of pyroptosis signaling-related molecules. Percentages of positively expressing cells are indicated. **b** Immunohistochemical detection of GSDMD and Casp1 in osteocytes from the SCB of OA knees. **c** Immunohistochemical detection of GSDMD in osteocytes from the SCB of knees in experimental OA models. Percentages of stained cells are shown in the right panels. **d** Percentage of empty lacunae in the SCB of knees in experimental OA models. **e** SCB plate thickness in the knees of the experimental ACLT-OA model. **f** Quantification of osteoclast number and size on the SCB surface. Left panels show TRAP-stained sections; right panels show osteoclast quantification. In bar charts, each symbol represents an individual sample, and bars with whiskers indicate the mean ± SEM. Statistical significance was determined using a two-tailed Student’s *t*-test. *P < 0.05; **P < 0.01; ***P < 0.001; ****P < 0.000 1. *ACLT* anterior cruciate ligament transection, *CIOA* collagenase-induced osteoarthritis

To initially assess pyroptosis induction in osteocytes within the subchondral bone (SCB), publicly available single-cell RNA-sequencing (scRNA-seq) data from SCB in OA knees were analyzed. Osteocytes were identified based on positive expressions of *SPARC* and *BGN* and a lack of *ALP*. Among the 976 osteocytes identified, 14.34% expressed *GSDMD*, 7.17% expressed *CASP1*, 21.31% expressed *CASP4*, and 47.13% expressed *IL-1β* (Fig. 1a). In line with these findings, immunostaining of clinical and experimental OA cartilage using specific antibodies against Casp1 and GSDMD revealed the presence of Casp1- and GSDMD-positive osteocytes in human OA knee samples (Fig. 1b). Notably, the number of positive osteocytes was significantly higher in experimental OA knees, both ACLT- and CIOA-OA models, compared to their relative controls (Fig. 1c, d). These results indicate the activation of pyroptosis signaling in osteocytes and increased bone remodeling in SCB bone during OA. Consequently, the SCB plate in the OA knees of experimental ACLT-OA model appeared thinner compared to control mice and showed an increased number of empty lacunae and TRAP-positive cells, suggesting a link between enhanced bone resorption and osteocyte death in the SCB region of OA knees (Fig. 1e, f).

**Fig. 2:**
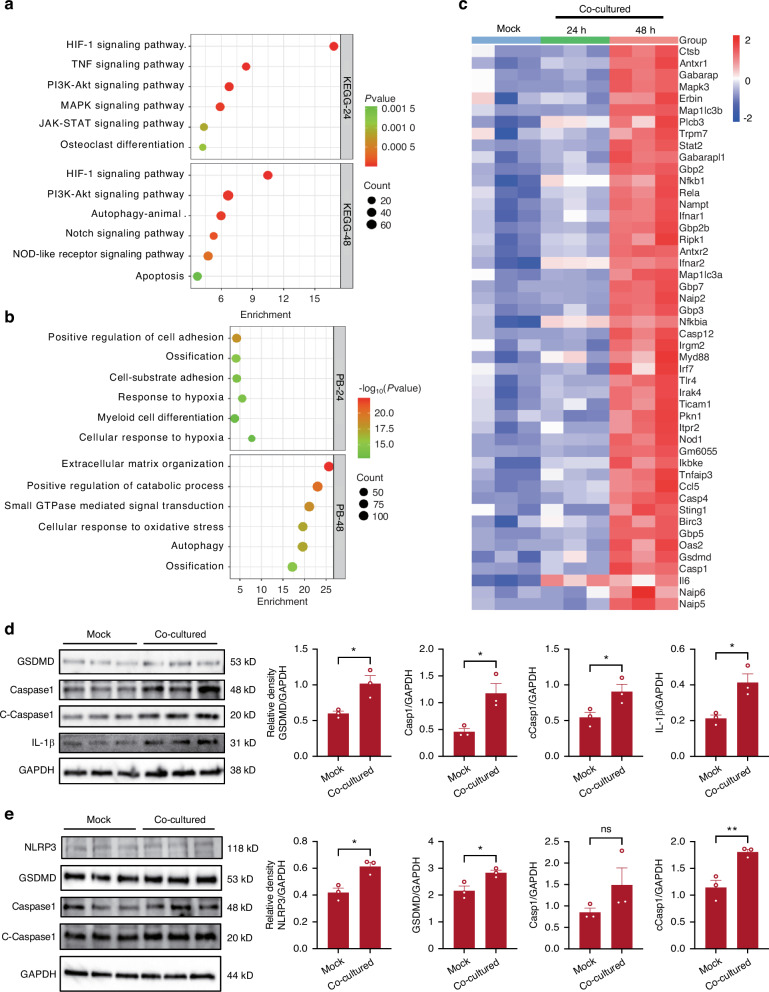
Detection of pyroptosis signaling in osteocytes co-cultured with stimulated chondrocytes. **a** KEGG pathway enrichment analysis of genes upregulated in MLO-Y4 cells following co-culture. **b** Gene Ontology analysis of the differentially upregulated genes. **c** A heatmap showing differentially expressed genes in the NOD-like receptor signaling pathway. **d** Western blot detection of pyroptosis markers in MLO-Y4 cells co-cultured with stimulated chondrocytes. Right panels show quantification of protein expression based on band intensity. **e** Western blot detection of pyroptosis markers in primary osteocytes co-cultured with stimulated chondrocytes. Right panels show quantification of protein expression based on band intensity. In bar charts, each symbol represents an individual sample, and bars with whiskers indicate the mean ± SEM. Statistical significance was determined using a two-tailed Student’s *t*-test. *P < 0.05; **P < 0.01; ***P < 0.001; ****P < 0.000 1

Given that increased mechanical and biochemical stimuli in osteoarthritic joints drive chondrocytes to release catabolic factors that significantly affect neighboring tissues, particularly osteocytes in the subchondral bone, we further investigated whether stimulated chondrocytes could induce pyroptosis in osteocytes using a co-culture model of chondrocytes and osteocytes. Primary murine chondrocytes were stimulated with IL-1β for 6 h and then co-cultured with MLO-Y4 osteocyte-like cells for 24 and 48 h (Fig. S1A). RNA-seq analysis of MLO-Y4 cells co-cultured with IL-1β-stimulated chondrocytes revealed upregulation of 990 genes at 24 h and 3 057 genes at 48 h (Fig. S1B). KEGG pathway analysis of these upregulated genes showed significant enrichment in pathways of osteoclast differentiation and the NOD-like receptor (NLR) signaling pathway (Fig. 2a, c). Additionally, Gene Ontology (GO) analysis indicated enrichment in biological processes such as response to hypoxia and cellular response to oxidative stress (Fig. 2b). Gene Set Enrichment Analysis further demonstrated that the upregulated genes in MLO-Y4 osteocytes co-cultured with IL-1β-stimulated chondrocytes were predominantly enriched in the NLR signaling pathway, with the majority involved in pyroptosis-related signaling (Fig. S1C). Western blot analysis confirmed the increased protein expression of Casp1, GSDMD and Il-1β in MLO-Y4 cells co-cultured with IL-1β-stimulated chondrocytes (Fig. 2d). In a manner analogous to that observed in MLO-Y4 osteocytes, activation of pyroptosis signaling was observed in primary osteocytes under the same co-culture condition (Fig. 2e). Together, these findings demonstrate that the pathological progression of OA involves enhanced SCB remodeling accompanied by activation of pyroptosis signaling in osteocytes. Moreover, increased expression of pyroptosis-associated molecules in osteocytes, including GSDMD, a protein classically recognized as an executor of pyroptosis, suggests its potential role in osteocytes of the SCB during OA.

### *Gsdmd* deletion in osteocytes aggravates OA progression via promoting bone remodeling in SCB

**Fig. 3:**
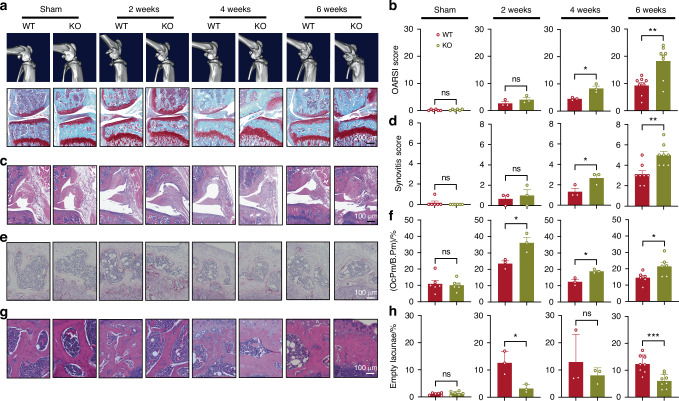
Pathological changes in cartilage and SCB following ACLT-induced OA in WT and *Gsdmd*-deficient mice. Analyses were performed at 2, 4, and 6 weeks post-surgery. **a** Representative 3D micro-CT images of knee joints at the indicated time points. Representative Safranin O-stained sections of distal femoral and proximal tibial cartilage. **b** OARSI cartilage scores. **c** Representative H&E-stained sections of synovium. **d** Synovitis scores. **e** Representative TRAP-stained sections of SCB. **f** Quantification of osteoclast-positive area on the bone surface. **g** Representative H&E-stained sections of SCB showing viable osteocytes and empty lacunae. **h** Quantification of empty lacunae in SCB. Scale bars are indicated. In bar charts, each symbol represents an individual sample, with bars and whiskers denoting the mean ± SEM. Statistical significance was assessed using a two-tailed Student’s *t*-test: *P < 0.05; **P < 0.01; ***P < 0.001; ****P < 0.000 1

**Fig. 4:**
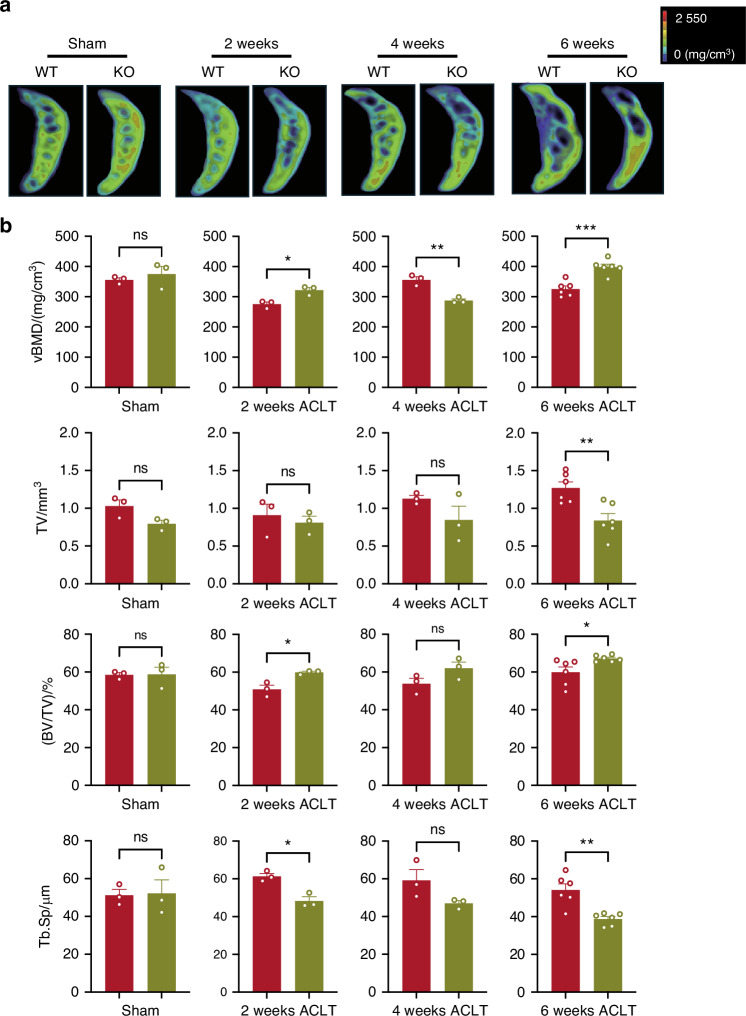
The SCB microarchitecture following ACLT-induced OA in WT and Gsdmd-deficient mice. **a** Representative micro-CT images of SCB in the proximal tibia. **b** Quantification of bone microarchitectural parameters, including vBMD, TV, BV/TV, and Tb.Sp. In bar charts, each symbol represents an individual sample, with bars and whiskers indicating the mean ± SEM. Statistical significance was determined using a two-tailed Student’s *t*-test: *P < 0.05; **P < 0.01; ***P < 0.001; ****P < 0.000 1

Our findings showing increased GSDMD expression in osteocytes raise the question of whether osteocyte-derived GSDMD functions pathologically or instead serves a homeostatic role in SCB during OA. To address this, we utilized osteocyte-specific *Gsdmd*-deficient mice in an ACLT-induced OA model. Knee joints were harvested at 2-, 4-, and 6-week post-surgery for pathological assessment. Notably, osteocyte-specific deletion of *Gsdmd* led to a more severe OA phenotype in the ACLT model, as evidenced by significantly higher OARSI cartilage degeneration scores, increased synovitis scores, and a greater number of TRAP-positive cells in *Gsdmd*-deficient mice compared with WT controls (Fig. 3a–f). Importantly, the number of empty lacunae in the SCB was significantly reduced in *Gsdmd*-deficient mice compared with WT mice beginning at 2 weeks post-surgery (Fig. 3g, h), indicating reduced osteocyte death. Increased osteophyte formation was observed in *Gsdmd*-deficient mice at 6 weeks post-surgery (Fig. S2). Additionally, micro-CT analysis revealed that *Gsdmd*-deficient mice showed a decrease in volumetric bone mineral density (vBMD) at week 4, followed by an increase at week 6 in the subchondral bone, compared to WT mice (Fig. 4a, b). BV/TV values were significantly higher in *Gsdmd*-deficient mice than in WT mice only at weeks 2 and 6, while trabecular separation (Tb.Sp) was lower at weeks 2 and 6. Consistently, the expression of bone turnover-related genes was significantly elevated in the SCB of *Gsdmd*-deficient mice, indicating increased bone remodeling (Fig. S3). These results suggest that OA subchondral bone changes reflect high-turnover remodeling rather than simple bone loss, which can ultimately lead to subchondral sclerosis characterized by increased BV/TV and BMD. Our findings indicate that osteocyte-specific deletion of *Gsdmd* leads to increased SCB remodeling and exacerbated cartilage degeneration in experimental OA, despite protection of osteocytes from cell death.

### *Gsdmd* deletion in osteocytes promotes bone remodeling and osteoporotic features in ovariectomized mice

**Fig. 5:**
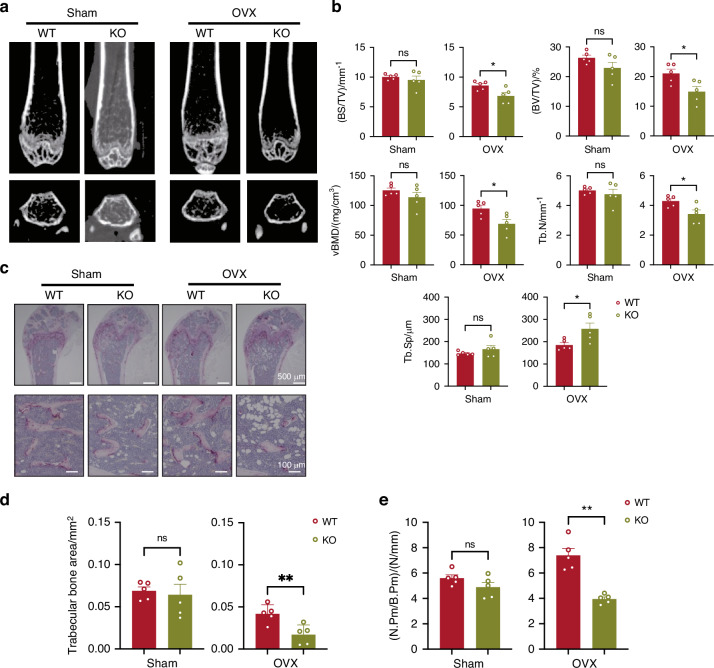
Osteoporotic features and pathological changes of femoral bones in WT and *Gsdmd*-deficient OVX mice. **a** Representative micro-CT images of femoral bones in OVX mice. **b** Quantification of bone parameters, including BS/TV, BV/TV, vBMD, Tb.N, and Tb.Sp. **c** Representative TRAP-stained sections of femoral bone, showing both primary and secondary spongiosa (scale bars indicated). **d** Quantification of trabecular bone area in the secondary spongiosa of the femur. **e** Quantification of TRAP-positive cells in the secondary spongiosa of the femur. In bar charts, each symbol represents an individual sample, with bars and whiskers indicating the mean ± SEM. Statistical significance was determined using a two-tailed Student’s *t*-test: *P < 0.05; **P < 0.01; ***P < 0.001; ****P < 0.000 1

To further gain additional evidence linking between deletion of *Gsdmd* in osteocytes and increased bone remodeling, osteocyte-specific *Gsdmd*-deficient mice were subjected to ovariectomy (OVX), and their femurs were collected for histomorphometric and histological analyses after 6 weeks. Micro-CT analysis revealed that *Gsdmd*-deficient mice exhibited significantly greater bone loss, as evidenced by reduced BV/TV, bone surface per tissue volume (BS/TV), vBMD, and trabecular number (Tb.N), along with increased Tb.Sp, compared to OVX-WT control (Fig. 5a, b). Histological analysis revealed a significant reduction in trabecular bone area and a decreased number of TRAP-positive cells in the secondary spongiosa of the femur in *Gsdmd*-deficient mice compared to OVX-WT controls, indicating a late stage of osteoporosis (Fig. 5c–e). Collectively, these findings suggest that deletion of *Gsdmd* in osteocytes enhances bone remodeling, resulting in increased bone turnover and osteoporotic changes. These results support our earlier observations that loss of osteocyte-derived GSDMD alters bone remodeling dynamics. Thus, the increased subchondral bone remodeling observed in the ACLT-induced OA model is consistent with a broader role for osteocyte-derived GSDMD in regulating bone turnover.

### Stimulation of *Gsdmd*-deficient osteocytes is associated with increased expression of inflammatory and bone remodeling mediators

**Fig. 6:**
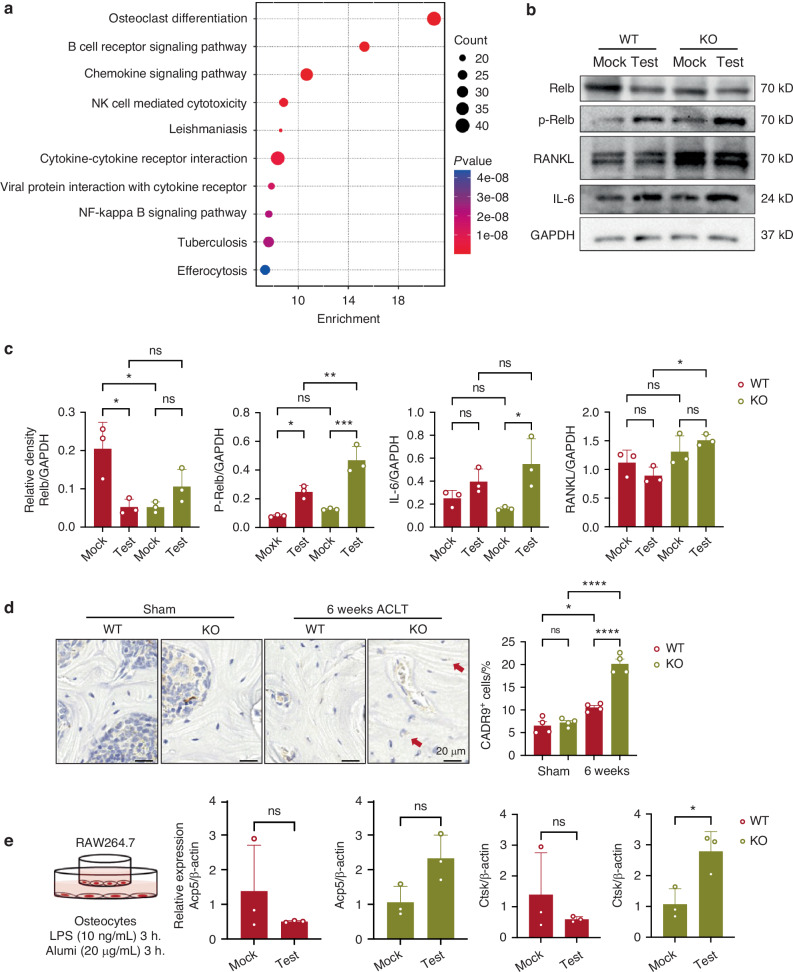
Molecular responses of *Gsdmd*-deficient osteocytes and their contribution to subchondral bone pathology. **a** KEGG pathway enrichment analysis of upregulated genes. **b**, **c** Western blot detection of inflammatory and bone turnover markers in primary osteocytes. **b** Representative images. **c** Quantification of the relative density of the band of each target to the reference molecule GAPDH. **d** Immunohistochemical detection of CARD9 in osteocytes from the SCB of ACLT-OA knees. Scale bars are indicated. Quantification of card9-positive cells in the right panel. **e** Schematic of the experimental setup showing the co-culture of stimulated osteocytes with RAW264.7 cells. The right panels for relative gene expression of osteoclast differentiation markers. In bar charts, each symbol represents an individual sample, and bars with whiskers indicate the mean ± SEM. Comparisons between two independent groups were performed using Student’s *t*-test, while differences among multiple groups were analyzed by one-way ANOVA, followed by Tukey’s multiple comparisons test. *P < 0.05; **P < 0.01; ***P < 0.001; ****P < 0.000 1

To investigate the molecular mechanisms underlying increased bone turnover in *Gsdmd*-deficient mice, osteocytes were isolated from both WT and *Gsdmd*-deficient mice. The cells were stimulated with lipopolysaccharide (LPS) and aluminum hydroxide to activate pyroptosis signaling, and their responses were analyzed using bulk RNA-sequencing (Fig. S4A). Compared to WT osteocytes, *Gsdmd*-deficient osteocytes exhibited 1 126 upregulated and 874 downregulated genes in response to stimulation (Fig. S4B). Pathway GO enrichment analyses of the upregulated genes revealed significant enrichment in immune-related processes and osteoclastogenesis. Specifically, the genes that are associated with the cytokine-cytokine receptor interaction and osteoclast differentiation (KEGG pathway), and the activation of the immune response (biological process), indicating enhanced inflammatory signaling (Fig. 6a, Fig. S4C). Notably, these genes were also significantly enriched in the NLR and NF-κB signaling pathways, both of which play central roles in activating inflammatory signaling and promoting the production of inflammatory and bone turnover mediators. Stimulated *Gsdmd*-deficient osteocytes exhibited an increased expression of inflammatory mediators, including Tnf, Il6, Mip1, Mip2, and Cxcl as well as bone turnover mediators such as Rankl and Light (Fig. S4D). Consistently, GESA analysis for KEGG pathway enrichment demonstrated that the upregulated genes in *Gsdmd*-deficient osteocytes were predominantly enriched in the osteoclast differentiation, NF-κB signaling and NLR pathways (Fig. S4D). Western blot analysis confirmed the increased protein level of pRelb, RANKL, and IL-6 in *Gsdmd*-deficient osteocytes (Fig. 6b, c). Among the most significantly upregulated genes in stimulated *Gsdmd*-deficient osteocytes, *Card9*, which is known as a key adapter protein mediating NLR signaling and downstream NF-κB-dependent transcription, was the most significantly upregulated gene in stimulated *Gsdmd*-deficient osteocytes. Consistently, the number of CARD9-positive osteocytes was markedly higher in the subchondral bone of *Gsdmd*-deficient mice compared with WT controls in experimental OA (Fig. 6d). To further validate these findings, *Gsdmd*-deficient and WT osteocytes were stimulated with LPS for 1 h, and their responses were compared. *Gsdmd*-deficient osteocytes exhibited elevated expression of CARD9 along with increased levels of inflammatory and bone remodeling mediators compared to WT osteocytes (Fig. S5). These results indicate that stimulation of *Gsdmd*-deficient osteocytes activates alternative signaling pathways, leading to increased production of inflammatory and bone remodeling mediators. To further elucidate the direct contribution of *Gsdmd*-deficient osteocytes on the development of pathological changes in the bone microenvironment, these stimulated *Gsdmd*-deficient osteocytes were co-cultured with osteoclast precursors (RAW264.7 cells), primary osteoblasts, or chondrocytes. Importantly, stimulated *Gsdmd*-deficient osteocytes exerted stronger pro-osteoclastogenic effects than WT osteocytes, as evidenced by increased expression of osteoclast differentiation markers in RAW264.7 cells (Fig. 6e). Likewise, stimulated *Gsdmd*-deficient osteocytes exerted inhibitory effects on the expression of the osteoblast functional marker Col1a1 compared with WT osteocytes (Fig. S6A). The effects of stimulated Gsdmd-deficient osteocytes on chondrocytes were largely comparable to those of WT osteocytes. Although a slight reduction in MMP3 expression was observed, no substantial changes were detected in other chondrocyte markers. These findings suggest that altered osteocyte-chondrocyte interactions contribute only minimally to the exacerbated OA pathology observed in Gsdmd-deficient mice (Fig. S6B). Instead, the aggravated OA phenotype is more likely driven by alterations in subchondral bone remodeling rather than direct effects on chondrocytes. Together, these results suggest that loss of *Gsdmd* in osteocytes disrupts their homeostatic function within the bone microenvironment, resulting in dysregulated bone turnover, potentially through impaired coupling between osteoclasts and osteoblasts. To further determine the functional involvement of CARD9 in mediating the pathological effects of stimulated *Gsdmd*-deficient osteocytes, CARD9 activity was suppressed using a combined CRISPR-Cas9-mediated gene silencing approach and pharmacological inhibition to achieve maximal functional inhibition. Inhibition of Card9 reduced activation of the NF-kB signaling pathway (pP105) observed in *Gsdmd*-deficient osteocytes (Fig. S7B). Moreover, suppression of Card9 signaling attenuated the upregulation of *Acp5*, an osteoclast mutation marker, in RAW264.7 cells induced by co-culture with stimulated *Gsdmd*-deficient osteocytes (Fig. S7C). Together, these findings indicate that *Gsdmd*-deficient osteocytes exhibit heightened responsiveness to stimulation and actively promote the production of inflammatory and bone remodeling factors through enhanced CARD9 activation, thereby contributing to increased bone turnover in the SCB of *Gsdmd*-deficient mice.

## Discussion

Emerging evidence indicates that subchondral bone remodeling plays a critical role in the degeneration of the overlying cartilage, as a reduction in bone mineral density compromises the integrity of the bone microarchitecture, leading to increased mechanical stress on the cartilage. This overload can result in microfractures at the cartilage-subchondral bone interface, which may disrupt the cartilage tissue barrier and promote vascular invasion into the cartilage. These changes contribute to thinning of the cartilage layer, resulting in increased stress on the tissue and thereby exacerbating its degeneration.^7,12^ Therefore, a deeper understanding of the pathological mechanisms driving subchondral bone remodeling could provide valuable insights and open new avenues for the development of targeted therapies. Osteocytes, which constitute the majority of bone cells, have been proposed to act as key regulators of subchondral bone remodeling by promoting inflammation and osteoclastogenesis. Importantly, pyroptosis-related signaling was detected in osteocytes within the SCB in both clinical specimens and experimental OA models, indicating a potential role for this pathway in OA. Given that GSDMD is a key executor of pyroptosis, the present study examined the role of osteocyte-derived GSDMD in regulating and modulating SCB homeostasis during OA progression.

Osteocyte-specific deletion of *Gsdmd* did not attenuate OA pathology but instead markedly exacerbated disease severity. *Gsdmd*-deficient mice exhibited pronounced cartilage degeneration and heightened subchondral bone inflammation despite suppression of pyroptosis, indicating that, in OA, GSDMD functions in osteocytes not merely as a mediator of inflammatory cell death but as a critical homeostatic regulator of SCB remodeling. Loss of this regulatory function accelerates disease progression by increasing bone turnover, leading to structural alterations in the SCB that elevate mechanical stress on the overlying cartilage and promote its degeneration. Our findings are consistent with recent studies highlighting that GSDMD exerts more diverse functions beyond the initiation of inflammation, particularly in maintaining tissue homeostasis and normal physiological processes. Indeed, increasing evidence from cell-type-specific *Gsdmd* deletion studies suggests that GSDMD is involved in regulating the function of multiple cellular compartments, including mitochondria, peroxisomes, endosomes, and the nucleus, where it acts as a mediator of organelle integrity and function.^10,13,14^ Specifically, GSDMD seems to be involved in maintaining bone mass, as specific deletion of *Gsdmd* in myeloid cells resulted in more severe bone loss in OVX- and aging-induced osteoporosis models.^15^ Authors have documented that GSDMD cleavage leads to the formation of p20 protein that regulates lysosomal maturation and secretion of osteoclasts. Other recent findings demonstrate that GSDMD-guided metabolite release by macrophages, which is essential for the repair process, as epoxyeicosatrienoic acid, a metabolite released via GSDMD pores, exhibits pro-repair activity and has the capacity to rejuvenate aged muscle.^16^ Specific deletion of *Gsdmd* in intestinal epithelial cells results in impaired mucus secretion and a concomitant loss of the mucus layer, accompanied by inefficient clearance of enteric pathogens at the mucosal surface.^17^ Likewise, GSDMD mediates the non-exosomal secretion of galectin-3, which is essential for the development of insulin resistance, as galectin-3 acts as an antagonist of insulin signaling.^18^ Therefore, molecules secreted through GSDMD pores may play important roles in cellular physiological processes such as efferocytosis, the phagocytic clearance of apoptotic cells, which is critical for maintaining tissue homeostasis. Disruption in the secretion of these molecules may impair efferocytosis by suppressing “find-me” and “eat-me” signals, resulting in the accumulation of apoptotic corpses and thereby subsequent promotion of chronic inflammation.^19^

Although *Gsdmd* deletion was associated with reduced osteocyte death, as indicated by fewer empty lacunae, this does not necessarily confer protection against OA. Empty lacunae likely represent only one aspect of OA-related subchondral bone alterations and do not directly reflect overall disease progression or severity.^20^ Indeed, deletion of *Gsdmd* in osteocytes was associated with altered cellular characteristics, rendering these cells more responsive to stimuli and leading to increased production of inflammatory mediators. This likely reflects the unique biology of osteocytes, which, unlike rapidly turning-over immune cells, are long-lived, terminally differentiated cells embedded within the mineralized matrix and thus poorly suited for extensive pyroptotic cell death. However, these findings are inconsistent with the conventional view of GSDMD as merely a pro-inflammatory factor or an executor of pyroptosis. GSDMD is widely recognized as a pore-forming effector molecule that mediates pyroptosis, an inflammatory form of cell death associated with the release of inflammatory cytokines and danger-associated molecular patterns, and has been implicated in a variety of inflammatory diseases. The pro-inflammatory nature of pyroptosis makes it an attractive therapeutic target for inflammatory diseases, including OA.^11^ Pyroptosis contributes to chondrocyte death and promotes extracellular matrix degradation, thereby accelerating cartilage degeneration. Consequently, targeting or genetically disrupting components of the pyroptosis signaling pathway has been shown to protect against OA progression by reducing inflammation and cartilage damage.^9,21,22^ Yang et al. reported that *Gsdmd* deficiency attenuates cartilage degeneration and synovitis in an OA model. However, the same study also showed that subchondral bone thickness was reduced in *Gsdmd*-deficient mice, despite the observed protection against cartilage degeneration and synovitis.^22^ The discrepancies between our findings and those reported by Yang et al. may be attributable to differences in the specificity of *Gsdmd* deletion, as our study employed osteocyte-specific *Gsdmd*-deficient mice, whereas their work utilized a global knockout model. Moreover, the therapeutic effects of pyroptosis inhibitors in experimental OA models may result from their action on other cell types, such as chondrocytes, macrophages, and synoviocytes, which directly contribute to cartilage degeneration and synovial inflammation.^9^ These findings together suggest that GSDMD exerts cell type-dependent functions within the joint, promoting cartilage degeneration and synovitis in certain cell populations, while serving a homeostatic guardian role in osteocytes to maintain subchondral bone homeostasis.

Our in vitro data demonstrated that chondrocytes activated NLRs in osteocytes via DAMPs, which trigger NF-κB, MAPK, and inflammasomes signaling involved in the production of pro-inflammatory cytokines (TNF and IL6) and induction of pyroptotic cell death. However, our findings suggest that the central driver of OA exacerbation is dysregulated inflammatory signaling in osteocytes, resulting from the loss of GSDMD. Dysregulation of NLRs signaling has been documented in human chronic inflammatory disorders, as genetic mutations in components of this signaling pathway have been reported, including NOD1 in atopic disorders, NOD2 in Crohn’s disease, CARD9 in inflammatory bowel disease, and NLRP3 for Muckle-Wells syndrome.^23–25^ In fact, there is a possibility that these mutations may result in gain- or loss-of-function of the affected molecules, leading to overactivation of cell death and NF-κB signaling associated with these diseases. Our data showed *Gsdmd*-deficient osteocytes exhibited heightened responsiveness to stimuli compared to WT cells, associated with increased expression of *Card9* and genes involved in the activation of NF-κB signaling and pro-inflammatory pathways. This enhanced responsiveness of osteocytes, which is characterized by elevated production of inflammatory cytokines, provides a plausible explanation for the increased SCB remodeling observed in deficient mice. However, the enhanced responsiveness of osteocytes may be mediated by the upregulated expression of CARD9, an adapter protein that links pattern recognition receptors to NF-κB and MAPK signaling pathways, thereby promoting the production of inflammatory cytokines.^26^ These results suggest that CARD9 may be a promising therapeutic target for OA, as it serves as a major regulator of the inflammatory cascade in subchondral bone.

**Fig. 7:**
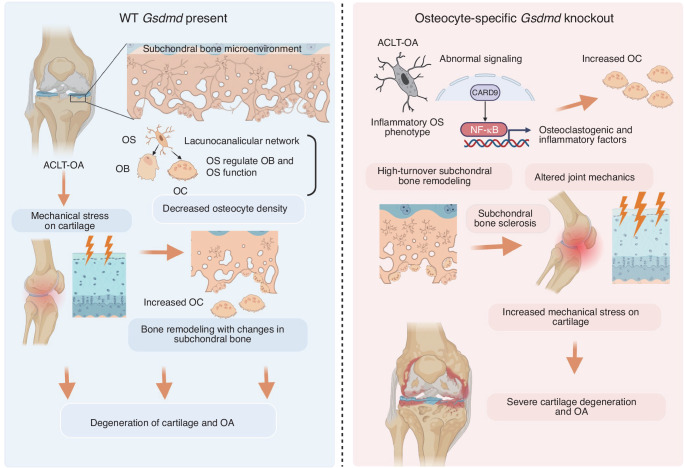
Proposed mechanism by which osteocyte-specific deletion of *Gsdmd* exacerbates OA progression. Osteocyte-specific deletion of Gsdmd leads to enhanced CARD9/NF-κB signaling and promotes an inflammatory osteocyte phenotype. This shift increases osteoclastogenic signaling, resulting in abnormal high-turnover SCB remodeling. The resulting structural alterations, including subchondral bone sclerosis, modify joint mechanical properties and increase mechanical stress on the overlying cartilage, thereby accelerating cartilage degeneration and OA progression. OS osteocyte, OB osteoblast, OC osteoclast

Although pyroptosis signaling in osteocytes can be triggered by various factors such as inflammation, trauma, and mechanical stress, our co-culture model demonstrated that stimulated chondrocytes promote pyroptosis signaling in co-cultured osteocytes, suggesting an additional pathway through which this form of cell death may be induced. The crosstalk between chondrocytes and osteocytes is facilitated by the structural continuity between articular cartilage and SCB, enabling reciprocal exchange of molecular signals and nutrients in response to mechanical and biological stimuli.^27^ Although diffusion represents a primary route for molecular and nutrient transport, increasing evidence has demonstrated the presence of porous channels and vascular structures that traverse the calcified cartilage, extend into the deep zone of articular cartilage, and penetrate the subchondral plate to connect with the marrow cavity, providing a morphological basis for bidirectional communication between cartilage and SCB.^28,29^ However, the observation that osteocyte-specific deletion of *Gsdmd* exacerbated OA pathology despite suppression of pyroptosis indicates that GSDMD’s function extends beyond the regulation of cell death. These findings can be explained by the loss of *Gsdmd*, which is associated with the development of a pro-inflammatory osteocyte phenotype characterized by impaired release of GSDMD-mediated factors and metabolites that normally regulate bone remodeling, thereby disrupting the osteocyte-centered regulatory network in the subchondral bone. This shift drives increased bone turnover through extensive crosstalk with osteoclasts and osteoblasts via the lacunar-canalicular network, leading to structural alterations in the subchondral bone microenvironment, including subchondral bone sclerosis, and, consequently, increased mechanical stress on the overlying cartilage, thereby exacerbating OA progression and cartilage degeneration (Fig. 7).

The major limitation of this study is that only male mice were used in the ACLT-induced OA model. However, in our OVX model, which utilized female mice, osteocyte-specific Gsdmd knockout mice similarly exhibited increased bone remodeling compared with WT mice. While this observation suggests that osteocyte-derived GSDMD may regulate bone turnover in both sexes, future studies are needed to determine whether osteocyte-derived GSDMD exerts comparable effects in female mice during OA progression, given the well-documented sex differences in OA pathophysiology.

In conclusion, our findings provide clear evidence for the critical role of osteocyte function in osteoarthritis, as disruption of osteocyte homeostatic regulation leads to increased SCB remodeling and contributes to disease progression. This study identifies GSDMD as a homeostatic guardian in osteocytes rather than a simple executor of pyroptosis, with its loss accelerating OA progression. Loss of this regulatory function promotes abnormal high-turnover remodeling of SCB, ultimately accelerating cartilage degeneration following joint injury. Although targeting GSDMD-mediated pyroptosis has demonstrated beneficial effects in experimental OA models, its clinical application should be approached with caution, as it may interfere with essential physiological processes or promote a pro-inflammatory phenotype in certain cell types. From a clinical perspective, individuals with gain-of-function mutations in GSDMD may exhibit an exaggerated inflammatory response in bone tissue when exposed to environmental stimuli, making them more susceptible to OA and other bone-related diseases.

## Materials and methods

### Study approvals and ethics

The protocols for the use of human samples in our experiments were approved by the Research Ethics Review Board of Hokkaido University Hospital with the consent of patients/donors (Approval No. 021-0029). The patients’ informed consent was obtained for use in this study. Animal experiments and genetic recombination experiments were conducted with the approval of the Hokkaido University Graduate School of Medicine Animal Experiment Facility Committee (21-0044, 2018-048).

### Human samples and immunohistochemistry (IHC) staining

Cartilage and subchondral bone were collected from late-stage OA patients who underwent total knee arthroplasty. Samples were fixed with 10% formalin (Wako, Osaka, Japan), declassified by treatment with EDTA (Wako, Osaka, Japan), embedded in paraffin, and finally sectioned into slices (3 μm thickness) for IHC staining. Sections were incubated with primary antibody targeting GSDMD (Cell Signaling, Massachusetts, USA; catalog no. #39754) and Caspase1 (GeneTex, California, USA; catalog no. GTX101322), and the signals were amplified with a horseradish peroxidase-conjugated specific antibody, followed by counterstaining with hematoxylin (Wako, Osaka, Japan).

### Isolation of primary murine osteocytes

Primary osteocytes were isolated from long bones of 7-week-old C57BL/6 mice (Clea, Tokyo, Japan), as earlier described.^30^ Briefly, Long bones of the extremities were dissected, epiphyses were cut off, and marrow cavities were carefully washed with PBS (NACALAI TESQUE, Kyoto, Japan; catalog no. 14249-95) to remove marrow cells using a needle and syringe. Bone samples were then cut into 1–2 mm fragments, followed by digestion with trypsin solution (Wako, Osaka, Japan; catalog no. 201-16945) for 20 min in 37 °C, 5% CO_2_ incubator. Thereafter, bone pieces were washed with PBS and digested with 0.001 g/mL collagenase type II solution (Gibco, Thermo Fisher Scientific, Massachusetts, USA; catalog no.17101015) at 37 °C for 1 h. Bone pieces were next suspended in EDTA solution and incubated at 37 °C for 30 min in a water bath with shaking. The EDTA solution was harvested, and bone pieces were further digested in 0.001 g/mL collagenase type I solution (Sigma-Aldrich, Missouri, USA; catalog no. C9891) at 37 °C water bath for 30 min with shaking. Collagenase type I solution was harvested, mixed with previously harvested EDTA solution, and centrifuged at 200 × *g* for 10 min. Repeated rounds of incubation with EDTA and collagenase type I solution were performed. Digested bone pieces were washed with PBS three times and cultured with isolated cells after 3rd time of digestion in α-minimum essential medium (Sigma-Aldrich, Missouri, USA; catalog no. M8042) supplemented with 5% fetal bovine serum (FBS) (Nichirei Biosciences, Tokyo, Japan; catalog no.1775012), 5% calf seraf (Sigma-Aldrich, Missouri, USA; catalog no. C8056) and 1% penicillin/streptomycin (Wako, Osaka, Japan; catalog no. 168-23191) at 37 °C with 5% CO_2_ in a humidified incubator for 7 days. The cells were identified as osteocytes by immunofluorescence. Briefly, cells were seeded on coverslips and then fixed by 4% paraformaldehyde solution (Wako, Osaka, Japan) for 30 min at room temperature. Cells were permeabilized with 1% Triton X-100 (Sigma-Aldrich, Missouri, USA) in PBS for 5 min and then incubated with the DMP1 antibody conjugated Alexa Fluor 488 (antibodies-online, Aachen, Germany; catalog no. ABIN6818494) diluted 1: 100 in 3% FBS in PBS for 2 h at 37 °C in a moist chamber. DAPI (Dojindo Molecular Technologies, Kumamoto, Japan; catalog no. D523) was used to label the nuclei of the cells. Coverslips were analyzed by an All-In-One Fluorescence microscope (BZ-X710, Keyence, Tokyo, Japan).

### Primary chondrocytes isolation

Primary articular chondrocytes were obtained from the cartilage of 5–6-day-old C57BL/6J mice, as previously described.^9^ Cartilage was carefully dissected from the femoral condyles and tibial plateau and subjected to overnight enzymatic digestion at 37 °C in DMEM (Sigma-Aldrich, Missouri, USA; catalog no. D5546) containing collagenase D (Roche Diagnostics GmbH, Mannheim, Germany; catalog no. 11088866001). The isolated cells were resuspended in DMEM supplemented with 10% heat-inactivated FBS, 2 mmol/L L-glutamine (Sigma-Aldrich, Missouri, USA; catalog no. G7513), and 25 mg/L penicillin/streptomycin solution. Experiments were performed using primary chondrocytes within the first 2 passages.

### Co-culture of osteocytes with stimulated chondrocytes

Primary murine osteocytes and MLO-Y4 osteocyte cell line (obtained from Professor Lynda F. Bonewald, Indiana Center for Musculoskeletal Health, ICMH, IU School of Medicine, Indianapolis, IN, USA) were seeded at a density of 1 × 10^5^ cells onto 24-well plates coated with 0.15 mg/mL collagen type 1 solution. On the other hand, primary murine chondrocytes were seeded (2 × 10^5^ cells) onto 1.0 µm cell culture transwell inserts (Falcon cell culture inserts, BD Biosciences, New Jersey, USA) and stimulated by treatment with 10 ng/mL interleukin-1β (IL-1β) (BioLegend, California, USA; catalog no. 575106) for 6 h. Thereafter, chondrocytes were washed with PBS 2 times and cultured for 24 and 48 h with seeded osteocytes onto a 24-well plate. Cells were subsequently harvested for further analysis.

### Generation of osteocyte-specific *Gsdmd*-deficient mice

Osteocyte-specific *Gsdmd*-deficient mice (*Gsdmd*^fl/fl^; DMP1-Cre) were generated using the Cre-loxP recombination system. Conditionally targeted *Gsdmd* floxed mice (*Gsdmd*^fl/fl^; RIKEN BRC, No. RBRC10762),^31^ in which critical exons of the *Gsdmd* gene are flanked by loxP sites, were crossed with DMP1-Cre transgenic mice (Lynda F. Bonewald, Indiana Center for Musculoskeletal Health, Indiana, USA),^32^ which express Cre recombinase specifically in osteocytes under the control of the dentin matrix protein 1 (DMP1) promoter. In the first generation (F1), *Gsdmd*^fl/+^; DMP1-Cre offspring were identified and further crossed with *Gsdmd*^fl/fl^ mice to generate *Gsdmd*^fl/fl^; DMP1-Cre mice (osteocyte-specific knockout). Genotyping was performed by PCR analysis using genomic DNA extracted from tail biopsies, with primers specific for the *Gsdmd* floxed allele and the Cre transgene. In *Gsdmd*^fl/fl^; DMP1-Cre mice, Cre-mediated recombination occurs specifically in osteocytes, resulting in targeted deletion of *Gsdmd* in these cells. Age- and sex-matched mice were used for all experiments. Furthermore, body weight and body length were measured and compared to those of WT mice at postnatal day 5 and at 4 weeks of age to assess the developmental status of *Gsdmd*-deficient mice. In addition, in 4-week-old mice, the bone mineral density of the distal femur was evaluated by μCT, and the thickness of the subchondral bone in the knee joint was assessed by Safranin O staining. No differences were observed in body length, body weight, or bone parameters between *Gsdmd*-deficient and WT mice (Fig. S8).

### Anterior cruciate ligament transection OA model (ACLT)

The 8-week-old C57BL/6J and *Gsdmd*-deficient male mice were used for the instability OA model. OA was induced in the left knee by dislocating the patella and cutting the anterior cruciate ligament with a microsurgical blade.^9^ The left knee joints were collected after 2, 4, and 6 weeks for histological examination. The joints were fixed in 10% formalin for 24 h, decalcified with EDTA, and then embedded in paraffin in a frontal orientation. The sections were subsequently stained with hematoxylin and eosin, as well as Safranin O-Fast green (Wako, Osaka, Japan). The histopathological evaluation was carried out using the Osteoarthritis Research Society International semiquantitative scoring system for cartilage. Additionally, synovitis and osteophyte formation were scored on the H&E-stained and Safranin O-stained sections, respectively.^33,34^ The subchondral bones of the tibial plateaus were further subjected to micro-CT (R_mCT2; Rigaku Corporation, Tokyo, Japan) analysis and bone histomorphometry analysis.

### Murine osteoporosis model

The 10-week-old C57BL/6J and *Gsdmd*-deficient female mice and osteocyte-specific knockout female mice were anesthetized by an intraperitoneal injection of 100 mg/kg of ketamine and 10 mg/kg of xylazine for bilateral ovariectomies.^35^ Mice were sacrificed 6 weeks after surgery, and their femurs were further subjected to micro-CT analysis and bone histomorphometry analysis.

### Bone morphometry

Femurs and knee joints of mice were harvested and fixed in 10% formalin for 24 h and then scanned by micro-CT at a 10 μm isotropic resolution, and X-ray energy was 80 kV and 80 mA. Parameters, such as the BS/TV, BV/TV, Tb.N, Tb.Sp, and vBMD were measured using a TRI/3D-BON (Ratoc System Engineering Co, Tokyo, Japan). For bone histomorphometric analyses, fixed samples were decalcified in EDTA and embedded in paraffin, and 5 μm-thick longitudinal sections were prepared and stained with TRAP (MilliporeSigma, Massachusetts, USA) with a methyl green counterstain to observe osteoclasts. The number of osteoclasts/bone surface (N.Oc/B.Pm) and size (Oc.Pm/B.Pm) at the secondary spongiosa of the distal femur or subchondral bone were determined using the ImageJ software. The area 250 μm proximal to the growth plate was defined as the primary spongiosa, and that of 250–1 000 μm proximal to the growth plate was defined as the secondary spongiosa.^36^

### Pyroptosis induction and co-culture models

Primary osteocytes isolated from C57BL/6J and *Gsdmd-*deficient mice were seeded on collagen type I-coated plates and stimulated with LPS (10 ng/mL) for 3 h, followed by aluminum hydroxide (20 µg/mL) for an additional 3 h to induce pyroptosis.^37,38^ Cells were then harvested for analysis. In the co-culture model, pyroptosis-induced osteocytes were seeded at a density of 1 × 10^5^ cells per well in 24-well plates and co-cultured with osteoclast precursors seeded onto transwell inserts for 24 h. RAW264.7 murine macrophage cell line (RIKEN BioResource Research Center, Ibaraki, Japan) was used as osteoclast precursors and seeded at a density of 3 500 cells per well, then cultured in medium containing recombinant mouse receptor activator of nuclear factor κB ligand (Biolegend, California, USA; catalog no. 462-TEC) (50 ng/mL) for 4 days before co-culturing. After co-culture, osteoclast precursors were subsequently harvested for further gene expression analysis. Using a similar approach, stimulated osteocytes were co-cultured for 24 h with either primary mouse osteoblasts or chondrocytes isolated from mice. Following co-culture, cells were harvested, and protein expression was evaluated by Western blotting.

### Combined genetic silencing and pharmacological inhibition of CARD9

Primary osteocytes isolated from WT-type and *Gsdmd-*deficient mice were seeded at a density of 1 × 10^5^ cells per well on collagen type I-coated 24-well plates. *Card9* silencing was achieved using the CRISPR-Cas9 system with CRISPRMAX™ Transfection Reagent (Thermo Fisher Scientific). Osteocytes were transfected with gRNA-containing medium and cultured for 48 h to induce *Card9* gene disruption. To achieve maximal inhibition of CARD9, osteocytes were subsequently pretreated with the selective CARD9 inhibitor BRD5529 (MedChemExpress) at a concentration of 20 μmol/L for 2 h. Cells were then stimulated with LPS (10 ng/mL) for 3 h in the continued presence of BRD5529 (20 μmol/L), followed by aluminum hydroxide stimulation for an additional 3 h, also in the presence of BRD5529. Following stimulation, cells were used for co-culture experiments.

### RNA isolation and quantitative real-time polymerase chain reaction (qRT-PCR)

In vitro, cells were homogenized and lysed using TRIzol (Invitrogen, California, USA; catalog no. 15596018). RNA was extracted using the NucleoSpin RNA Kit (Takara Bio, Shiga, Japan; catalog no. U0955C) and purified 0.25 μg RNA samples were used to synthesize cDNAs using the Go Script reverse transcriptase kit (Promega Corporation, Wisconsin, USA; catalog no. A5003). For extracting RNA from subchondral bone, the knee joints of mice were harvested after 4 weeks of the ACLT surgery. The surrounding muscles and ligaments, the distal femur and proximal tibia ends were exposed, articular cartilage was excised with a scalpel, and the bone ends distal to the growth cartilage and subchondral bone were excised. The extracted bone ends were split in half, and the bone marrow was washed off with PBS using a syringe. The harvested subchondral bone tissues were crushed using a Power Masher II (Nippi, Tokyo, Japan) under cooling with liquid nitrogen. Tissues were lysed using TRIzol and then subjected to RNA extraction and cDNA synthesis. The cDNAs were next assayed using the TB Green Premix Ex Taq II (Takara Bio, Shiga, Japan; catalog no. RR830L) with gene-specific primers listed in Table S1. Gene expression of each target was determined by the 2^−ΔΔCt^ method.

### Western blotting

Cells were lysed in EzApply sample buffer (ATTO, Tokyo, Japan; catalog no. AE-1430) and then heated at 100 °C for 5 min. The proteins extracted from the lysate were separated by SDS-PAGE and subsequently transferred onto PVDF membranes (Immobilon-P; Millipore, Massachusetts, USA; catalog no. IVPH00010) using an electrophoretic transfer system. The membranes were blocked with 3% skim milk and then incubated with primary antibodies at the concentrations specified by the manufacturers (Table S2). The secondary antibody conjugated with HRP respective primary antibody was used for the detection of bound antibodies. Signals were detected using Ez WestLumi Plus (ATTO, Tokyo, Japan; catalog no.WSE-7120), and the images were analyzed using an iBright 1500 Imaging System (Thermo Fisher Scientific, Massachusetts, USA). Band intensities were quantified relative to GAPDH levels using ImageJ.

### Bulk RNA-seq analysis

Libraries were constructed from 1 µg of high-quality RNA using the NEBNext Ultra II Directional RNA Library Prep Kit (New England Biolabs) and then sequenced using an Illumina NovaSeq X plus (Illumina, San Diego, CA, USA). The resulting data comprised 150 bp paired-end reads, yielding 6 G bases per sample, and roughly 26.7 million reads per sample were trimmed and aligned to the mouse reference genome (mm39) using HISAT2. Read counts were generated with FeatureCounts v2.0.6, and gene expression was quantified as fragments per kilobase of transcript per million mapped reads (FPKM) based on each gene’s length and the read counts.^39,40^ The reads were quantified via RSEM, and the raw count data were subjected to the edgeR (R package v3.22.5) to determine the differentially expressed genes and fold changes. Significance was set up on the basis of a <0.05 false discovery rate. GO and pathway enrichment analyses were performed via the public database for the gene-set enrichment tool ShinyGO (http://bioinformatics.sdstate.edu/go74/), and visualization/graphing was performed via SRplot tools (http://www.bioinformatics.com.cn/srplot).^39,40^ BBrowserX software (BioTuring Inc., San Diego, CA, USA: https://bioturing.com/bbrowserx) was used for analyzing single-cell RNA-sequencing (scRNA-Seq) public database (GSE196678).^41^ The RNA-seq data reported in this study are deposited in the Gene Expression Omnibus database (https://www.ncbi.nlm.nih.gov/geo/) with the accession numbers GSE301329 and GSE305276.

### Statistical analysis

No statistical methods were used to predetermine sample size. All statistical analyses were conducted using GraphPad Prism version 10.4.1 (Dotmatics, California, USA). Comparisons between two independent groups were evaluated using Student’s *t*-test, while differences among multiple groups were analyzed by One-way ANOVA, followed by Tukey’s multiple-comparison procedure. *P* value of less than 0.05 was deemed statistically significant.

## Supplementary information

Supplemental materials

Raw data

## Data Availability

All supporting data values are provided in the Supporting Data Values file. The RNA-Seq data included in this study are publicly available at the NCBI GEO database (https://www.ncbi.nlm.nih.gov/geo/) with accession numbers GSE301329 and GSE305276. Other materials can be obtained by contacting the corresponding author.
