# Supplementary material for: Osteocyte-specific gasdermin D deletion accelerates osteoarthritis via promoting subchondral inflammation and remodeling
Source: Bone Res. 2026 Jul 28;14:75. doi: 10.1038/s41413-026-00566-w (PMC13415536; doi:10.1038/s41413-026-00566-w)
Supplement: Supplementary file 1 — Supplemental materials [file 41413_2026_566_MOESM1_ESM.docx]

**Supplementary Materials**

**Osteocyte-specific Gasdermin D deletion accelerates osteoarthritis via promoting subchondral inflammation and remodeling**

Yuki Ogawa^1^, Taku Ebata^1^, Taiki Tokuhiro^1^, Liyile Chen^1^, Ryota Suzuki^1^, Yuki Fujie^1^, Masaya Nakajo^1^, Tsutomu Endo^1^, Hend Alhasan^1^, Masanari Hamasaki^1^, Daisuke Takahashi^1^, Koji Iwasaki^1^, Ken Kadoya^1^, Tomohiro Onodera^1^, Norimasa Iwasaki^1^, M Alaa Terkawi^1^*

^1^ Department of Orthopaedic Surgery, Faculty of Medicine and Graduate School of Medicine, Hokkaido University, Kita-15, Nishi-7, Kita-ku, Sapporo, Japan.

*Corresponding author: M Alaa Terkawi

Email: materkawi@med.hokudai.ac.jp

**8 Supplementary Figures**

**2 Supplementary Tables**


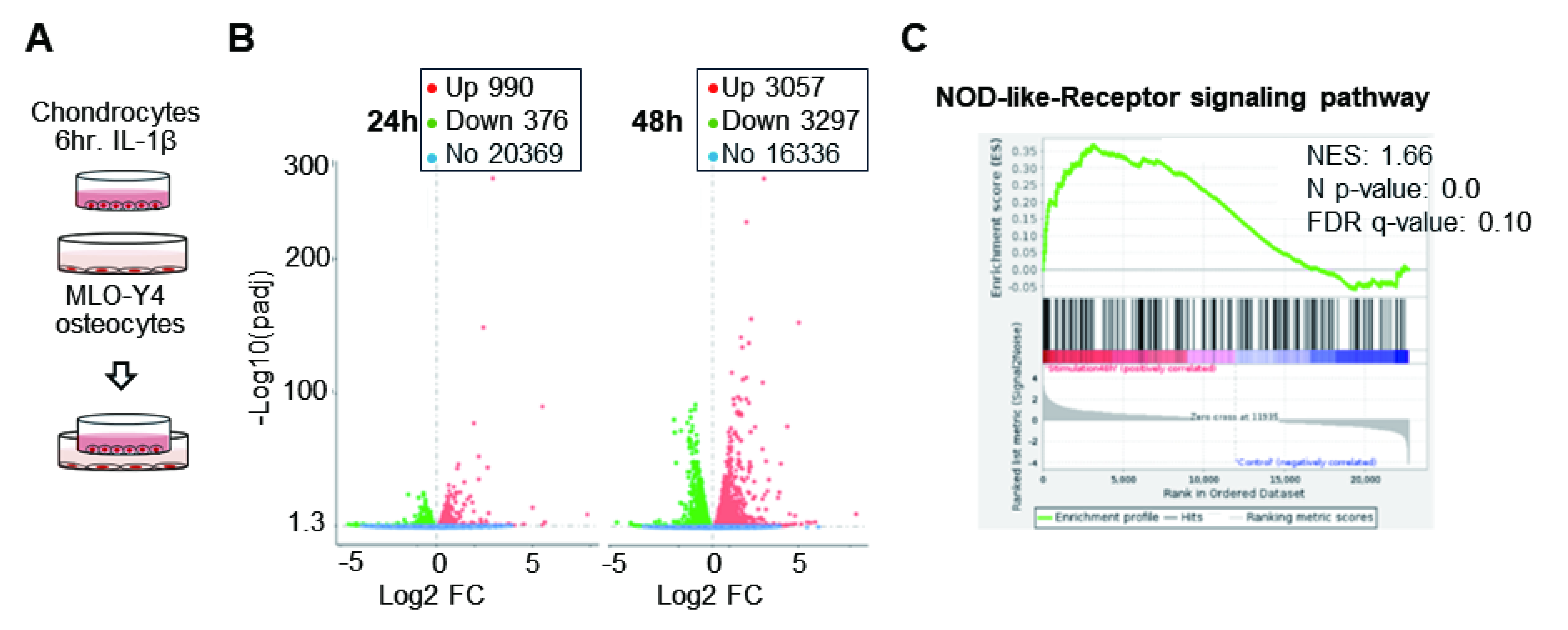


**Supplementary Figure 1. Detection of pyroptosis signaling in osteocytes co-cultured with stimulated chondrocytes.** A) Schematic of the experimental setup showing the co-culture of chondrocytes and MLO-Y4 osteocyte-like cells for 24 h and 48 h. B) Volcano plots depicting the molecular response of MLO-Y4 cells after co-culture with IL-1β-stimulated chondrocytes at 24 h and 48 h. C) GSEA analysis of the NOD-like receptor signaling pathway based on differentially expressed genes after 48 h of co-culture.


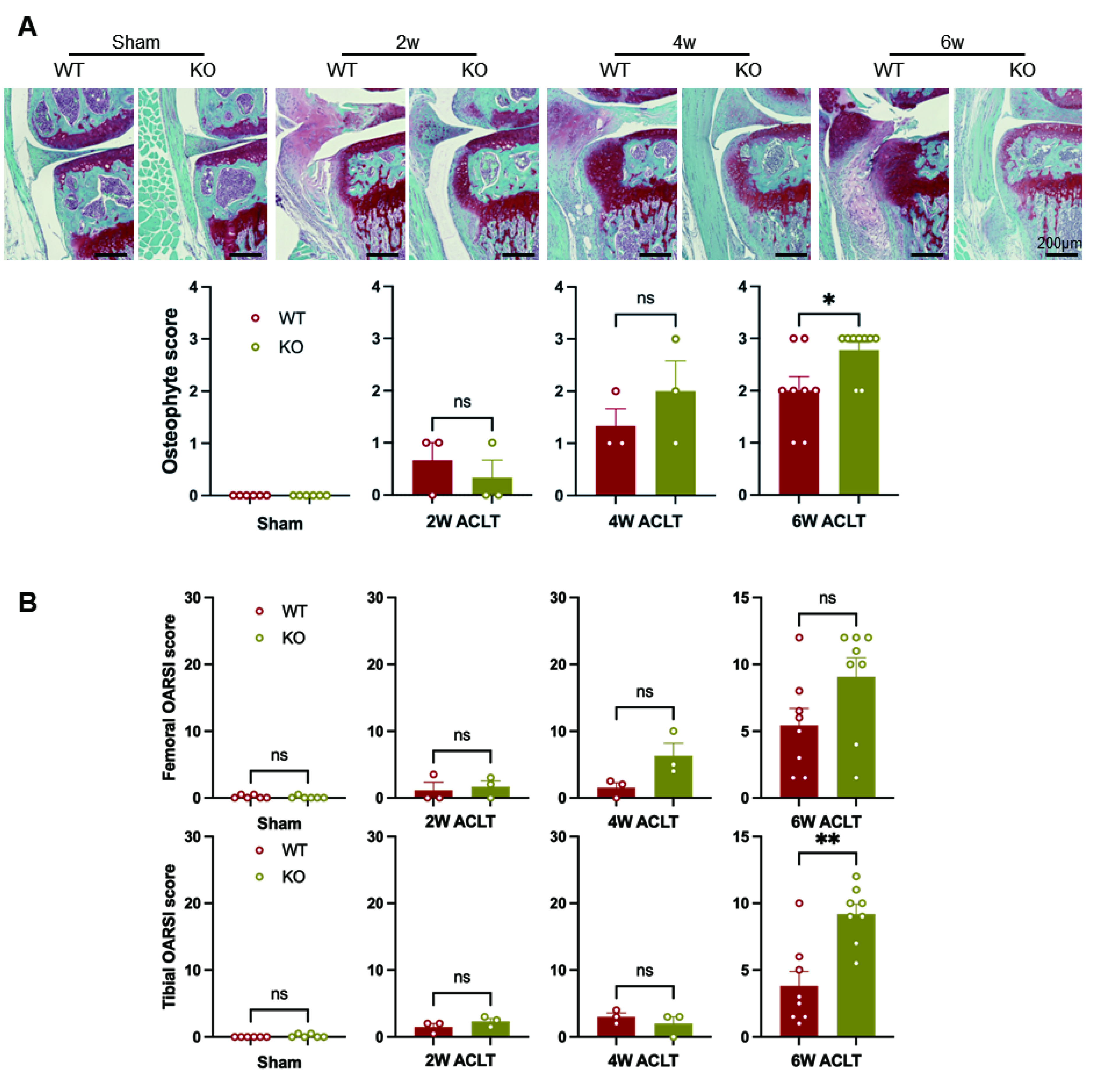


**Supplementary Figure 2. Osteophyte scores following ACLT-induced OA in knees of WT and *Gsdmd*-deficient mice.** A) Analyses were performed at 2, 4, and 6 weeks post-surgery. Upper panels, representative Safranin O-stained sections of cartilage. Scale bars are indicated. Lower panels, Quantification of osteophyte scores. B) Changes in femoral and tibial OARSI cartilage scores following ACLT-induced OA in WT and *Gsdmd*-deficient mice. In bar charts, each symbol represents an individual sample, with bars and whiskers denoting the mean ± SEM. Statistical significance was assessed using a Student’s t-test: *P < 0.05.


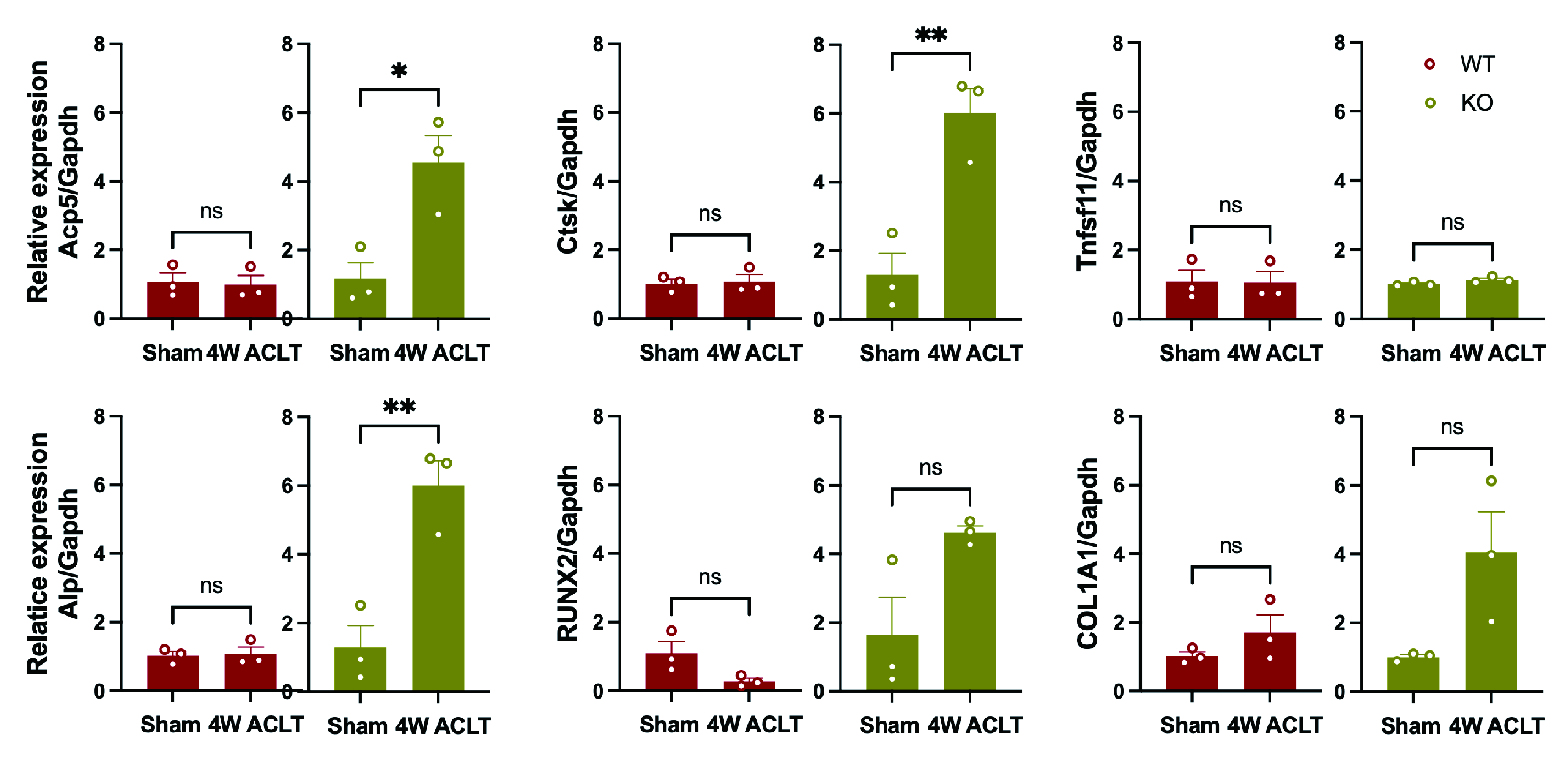


**Supplementary Figure 3. Relative gene expression of born turnover markers in subchondral bone tissue following ACLT-induced OA in WT and *Gsdmd*-deficient mice.** Analyses were performed at 4 weeks post-surgery using qRT-PCR. In bar charts, each symbol represents an individual sample, with bars and whiskers denoting the mean ± SEM. Statistical significance was assessed using a two-tailed Student’s t-test: *P < 0.05; **P < 0.01.


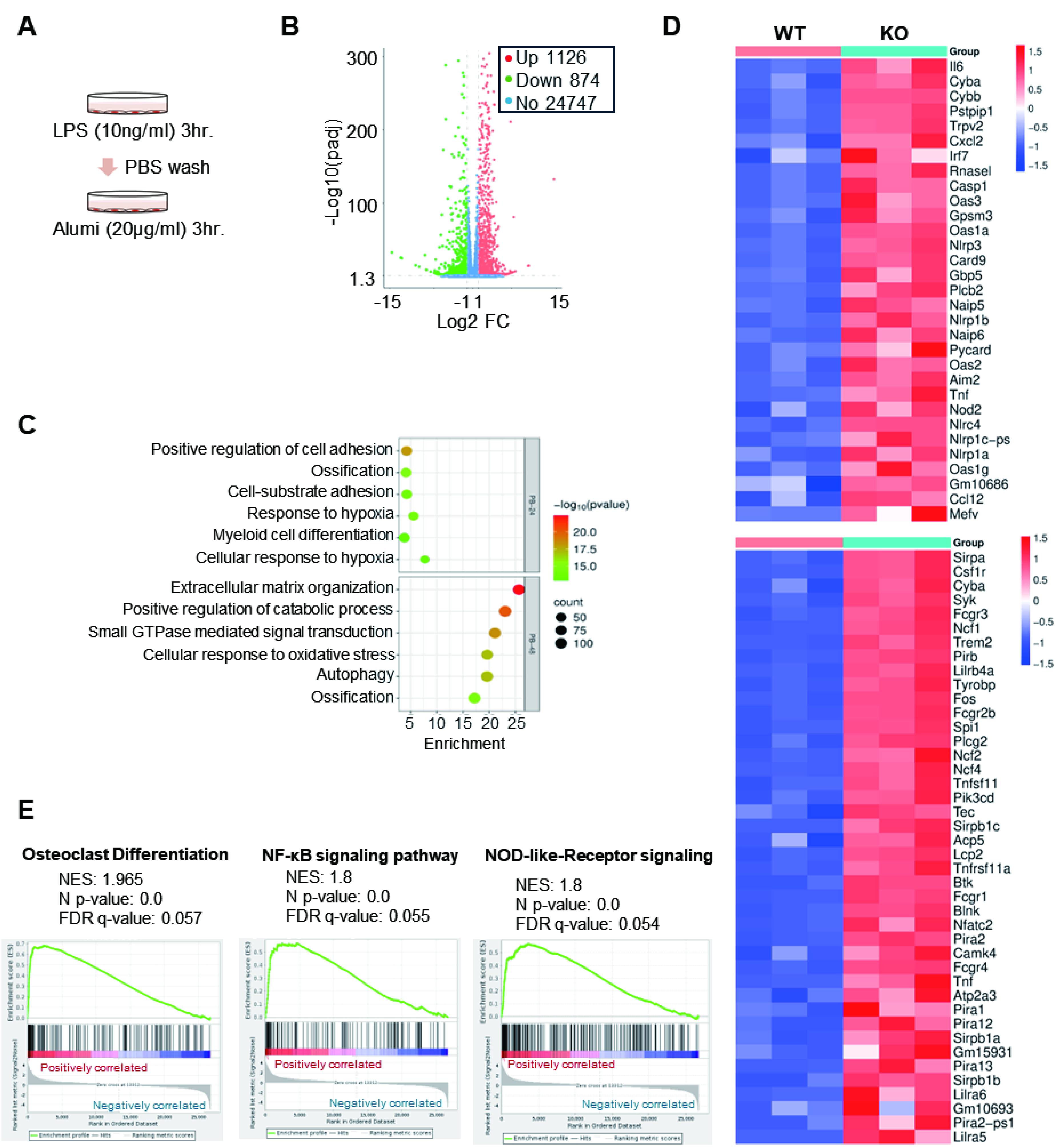


**Supplementary Figure 4. Molecular responses of *Gsdmd*-deficient osteocytes and their contribution to subchondral bone pathology.** A) Schematic of the experimental setup showing the stimulation condition to induce pyroptosis in osteocytes. B) Volcano plots depicting the molecular response of *Gsdmd*-deficient osteocytes KO as compared to WT osteocytes following stimulation with LPS and Alumi. C) Gene Ontology analysis of the differentially upregulated genes. D) Heatmap illustrating differentially expressed genes involved in the NOD-like receptor signalling in the upper panel and Osteoclast differentiation signalling pathways in the lower panel. E) GSEA analysis of the osteoclast differentiation, NF-κB signaling pathway, NOD-like receptor signaling pathway based on differentially expressed genes.


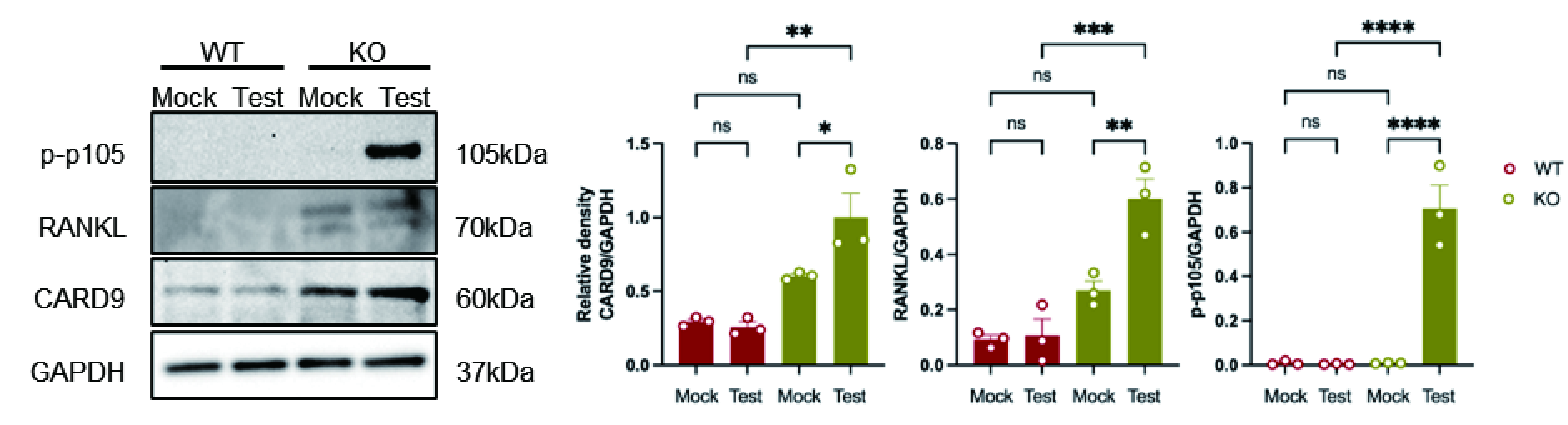


**Supplementary Figure 5. Quantification of relative band density of each target protein normalized to GAPDH in osteocytes from WT and *Gsdmd*-deficient mice after 1-hour LPS stimulation.** Left panels are representative blot images, and the right panels are quantification of band density of each target. In bar charts, each symbol represents an individual sample, with bars and whiskers denoting the mean ± SEM. Statistical significance was assessed using a one-way analysis of variance. *P < 0.05; **P < 0.01; ***P < 0.001; ****P < 0.0001.


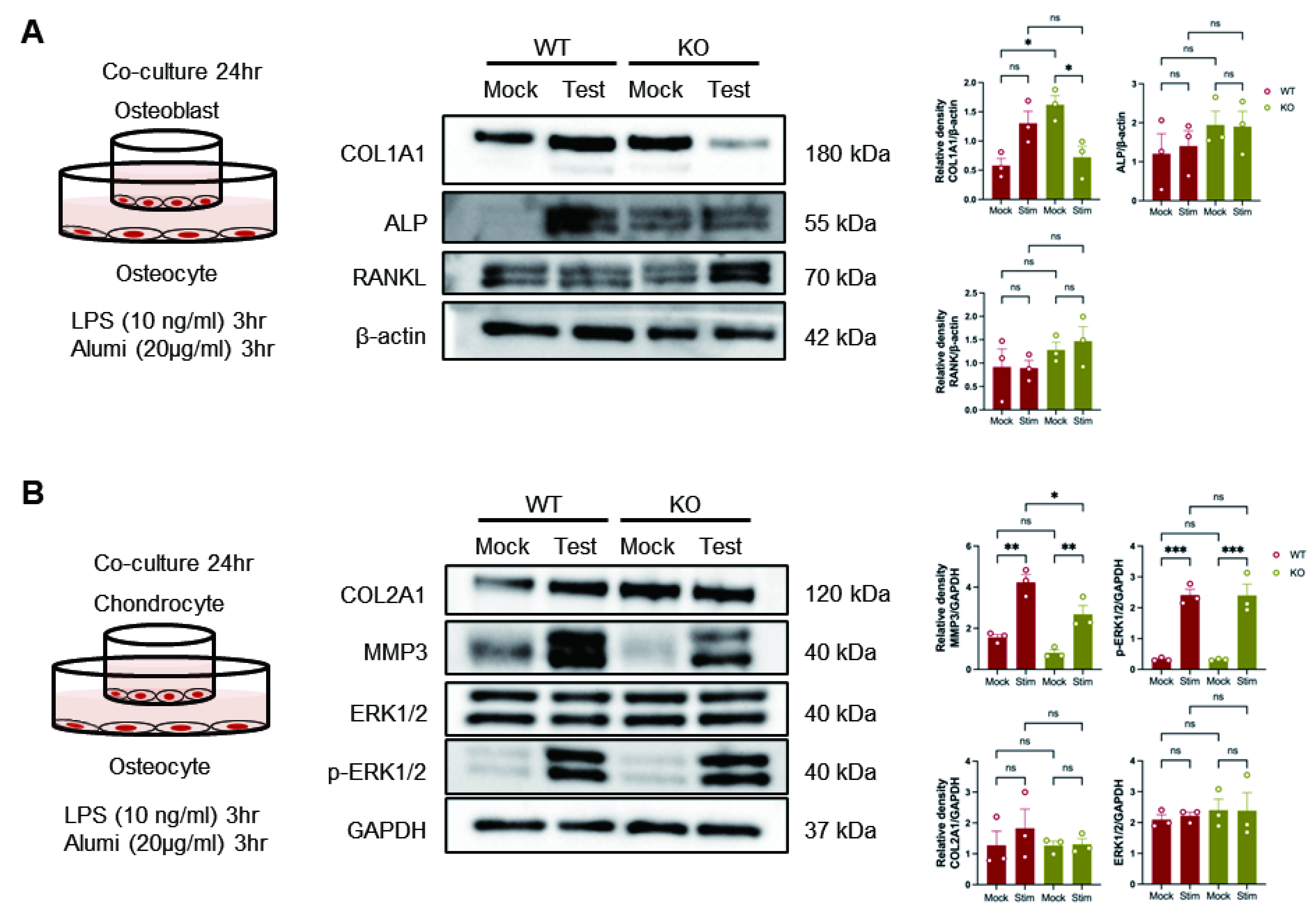


**Supplementary Figure 6.** **Quantification of relative band density of each target protein** **in chondrocyte or osteoblast after co-cultured. for 24 hours with LPS and Alumi stimulation osteocytes.** A) Schematic of the experimental setup showing the co-culture of stimulated osteocytes with osteoblast cells. Western blot detection of functional markers in osteoblast cells co-cultured with stimulated osteocytes. Right panels show quantification of protein expression based on band intensity. B) Schematic of the experimental setup showing the co-culture of stimulated osteocytes with chondrocyte cells. Western blot detection of anabolic and catabolic factor markers in chondrocyte cells co-cultured with stimulated Osteocytes. Right panels show quantification of protein expression based on band intensity. * = *P* < 0.05; ** = *P* < 0.01; *** = *P* < 0.001.


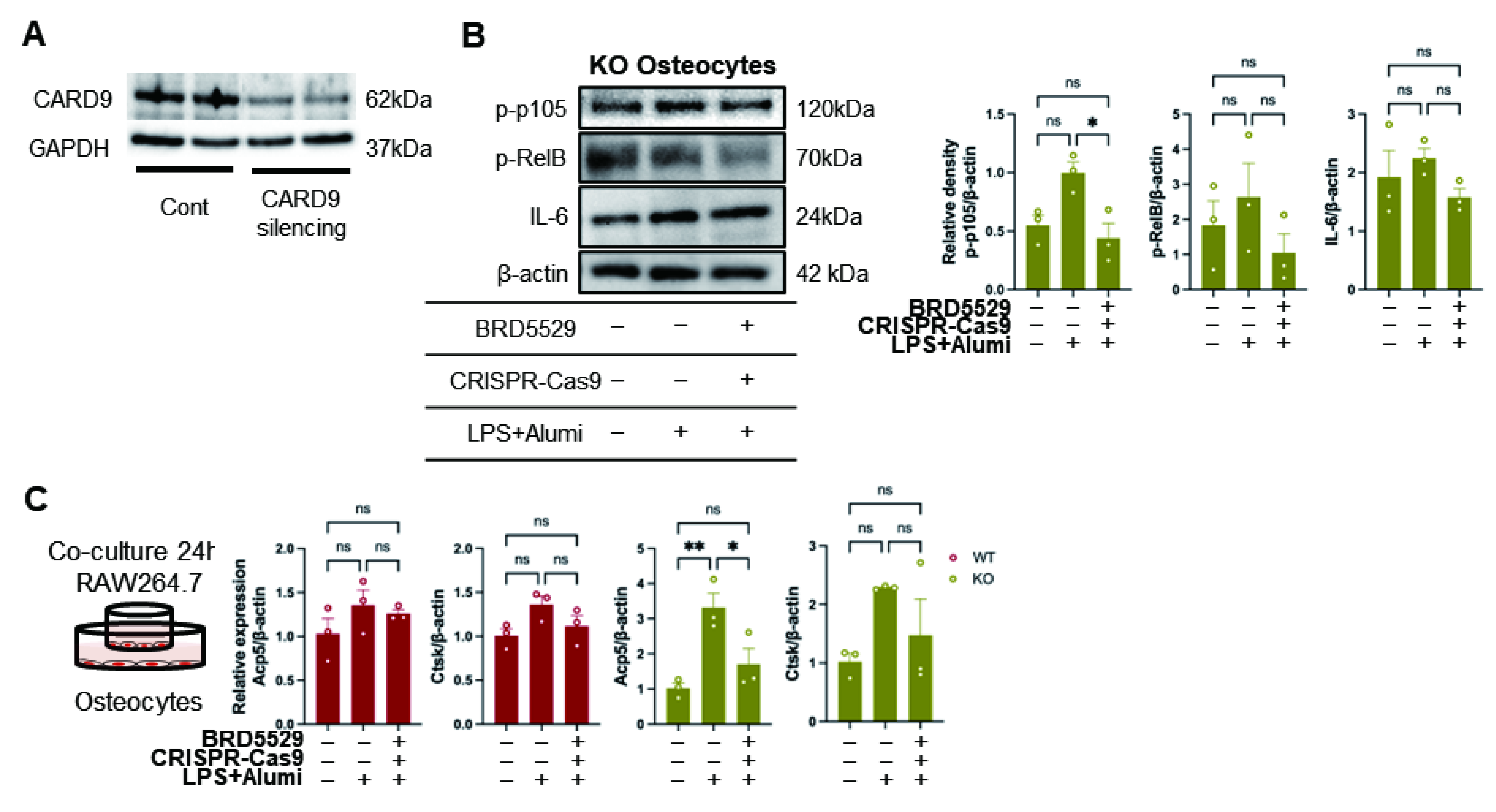


**Supplementary Figure 7. Effects of Card9 inhibition in osteocytes on osteoclast regulation in response to stimulation.** A) Western blot detection of. card9 in osteocytes after card9 silencing and inhibition. B) Western blot detection of NF-kB signaling pathway markers in osteocytes after stimulation with or without card9 silencing and inhibition. Right panels show quantification of protein expression based on band intensity. C) Schematic of the experimental setup showing the co-culture of stimulated osteocytes with RAW264.7 cells. The right panels for relative gene expression of osteoclast differentiation markers. * = *P* < 0.05; ** = *P* < 0.01.


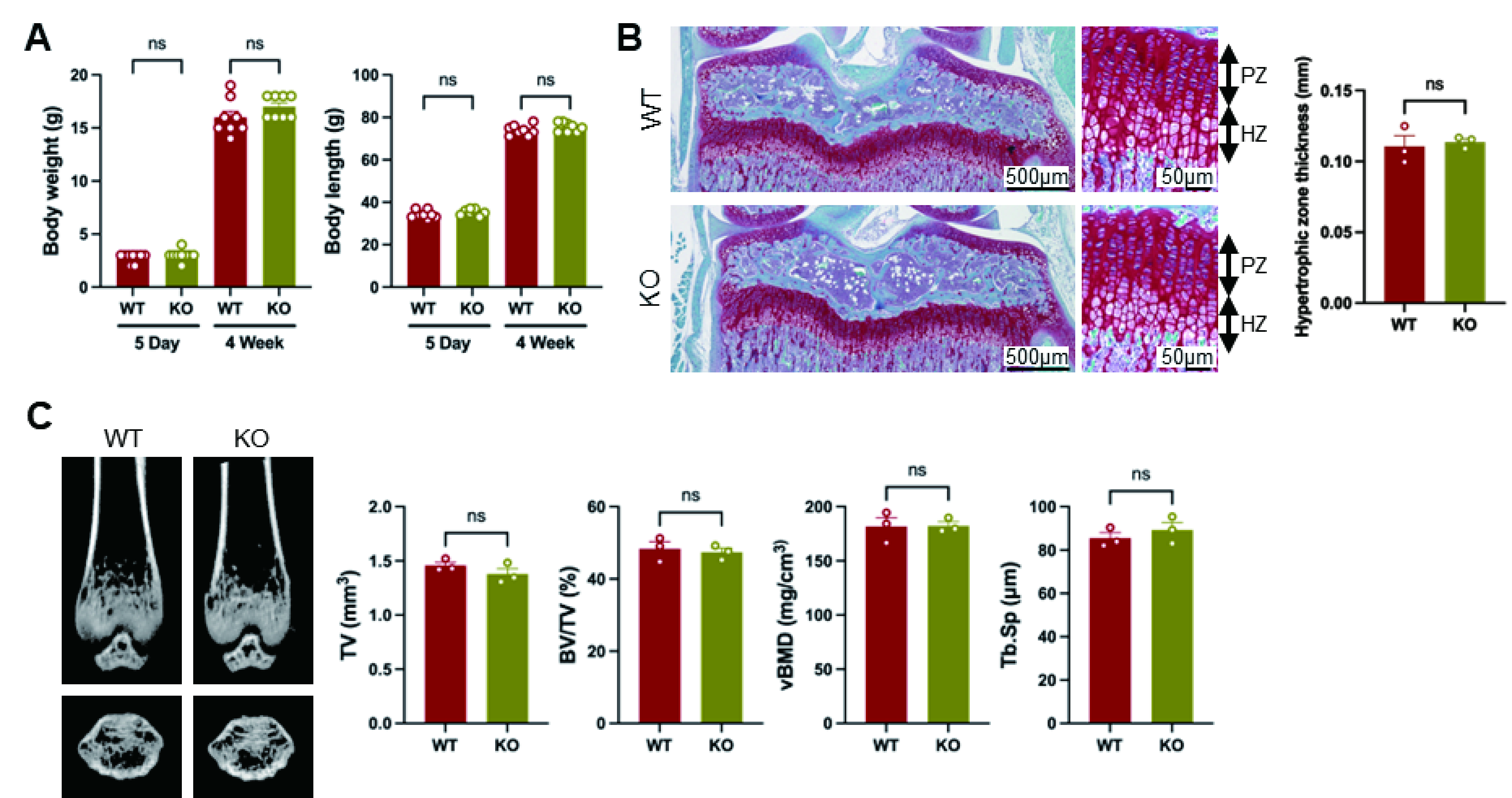


**Supplementary Figure 8.** **Skeletal development and growth plate in WT and KO mice** A) Body weight and body length measured at postnatal day 5 and 4 weeks of age in wild type (WT) and osteocyte specific *Gsdmd* knockout (KO) mice. B) Representative Safranin O staining sections of the proximal tibial growth plate from WT and KO mice. The right panel shows the hypertrophic zone thickness in each mouse group. Hypertrophic zone thickness was calculated as the ratio of the hypertrophic zone area to the growth plate width. PZ; proliferating zone, HZ; hypertrophic zone.  C) Representative micro-CT images of femoral bones in WT and KO mouse. The right panel shows quantification of bone parameters, including TV, BS/TV, vBMD, Tb.Sp.

**Supplementary Table 1.** **Primer list used in this study.**

| **Target** | **Forward** | **Reverse** |
| --- | --- | --- |
| ***mGapdh*** | TGCAGCGAACTTTATTGATG | ACTTTGTCAAGCTCATTTCC |
| ***mβ-actin*** | GGCTGTATTCCCCTCCATCG | CCTTCTGACCCATTCCCACC |
| ***mTnfsf11*** | AGGGAGCACGAAAAACTGGT | CGGAGCTTGAAAAATCCCCC |
| ***mRUNX2*** | TCTCCAGGAGGACAGCAAGA | GCAGCCTTAAATGACTCTGTTGG |
| ***mAlp*** | CCGGCTGGAGATGGACAAAT | GGACCTGAGCGTTGGTGTTA |
| ***mCOL1A1*** | ACCTTCCTGCGCCTAATGTC | GCTACGCTGTTCTTGCAGTG |
| ***mAcp5*** | TGTGGGCTATGTGCTGAGTG | GGACCTTTCGTTGATGTCGC |
| ***mCtsk*** | CAGAAGCAGTATAACAGCAAGGT | CCCAAATTAAACGCCGAGAGA |

| **Antibody** | **Dilution** | **Manufacturer** | **Research Resource Identifier (RRID)** |
| --- | --- | --- | --- |
| ***IL-1β*** | 1:2000 | Cell Signaling | AB_2793298 |
| ***NLRP3*** | 1:2000 | GeneTex | AB_2887025 |
| ***Caspase 1*** | 1:2000 | GeneTex | AB_10618781 |
| ***GSDMD*** | 1:2000 | Cell signaling | AB_2799760 |
| ***GAPDH*** | 1:10000 | BioLegend | AB_2734502 |
| ***Relb*** | 1:2000 | GeneTex | AB_11163446 |
| ***pRelb*** | 1:2000 | Thermo Fisher Scientific | AB_2896968 |
| ***RANKL*** | 1:2000 | Bioss | - |
| ***IL-6*** | 1:2000 | GeneTex | AB_10721789 |
| ***Card9*** | 1:2000 | GeneTex | AB_1949833 |
| ***Pp105*** | 1:2000 | Cell signaling | AB_2282911 |
| ***SOST*** | 1:1000 | Thermo Fisher Scientific | - |
| ***pSTAT3*** | 1:2000 | Cell signaling | AB_2491009 |
| ***Anti-rabbit HRP*** | 1:2000 | Cell signaling | AB_2099233 |
| ***Anti-rat HRP*** | 1:2000 | Cell signaling | AB_10694715 |
| ***Anti-mouse HRP*** | 1:2000 | Cell signaling | AB_330924 |

**Supplementary Table 2.** **Antibodies list used in this study.**
