# Supplementary material for: Osteocyte-specific gasdermin D deletion accelerates osteoarthritis via promoting subchondral inflammation and remodeling
Source: Bone Res. 2026 Jul 28;14:75. doi: 10.1038/s41413-026-00566-w (PMC13415536; doi:10.1038/s41413-026-00566-w)

Western blotting bands in Figure 2G.

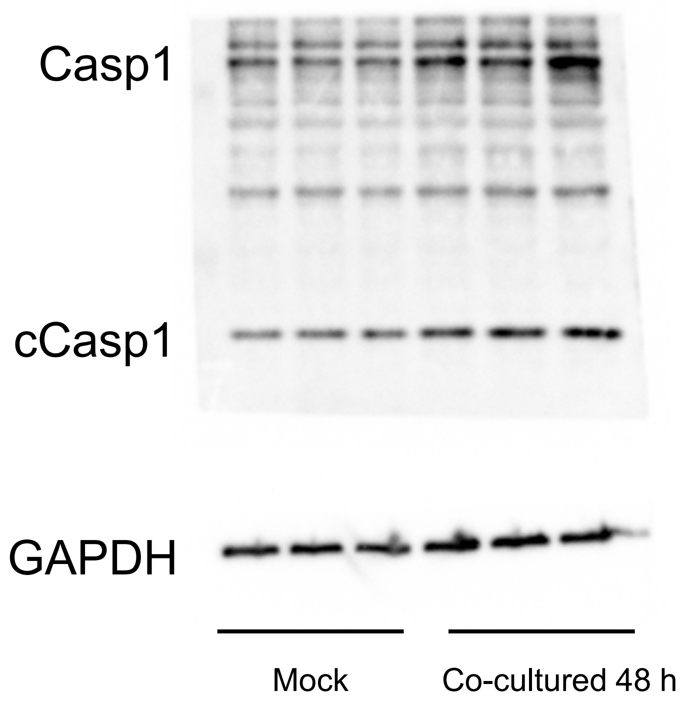

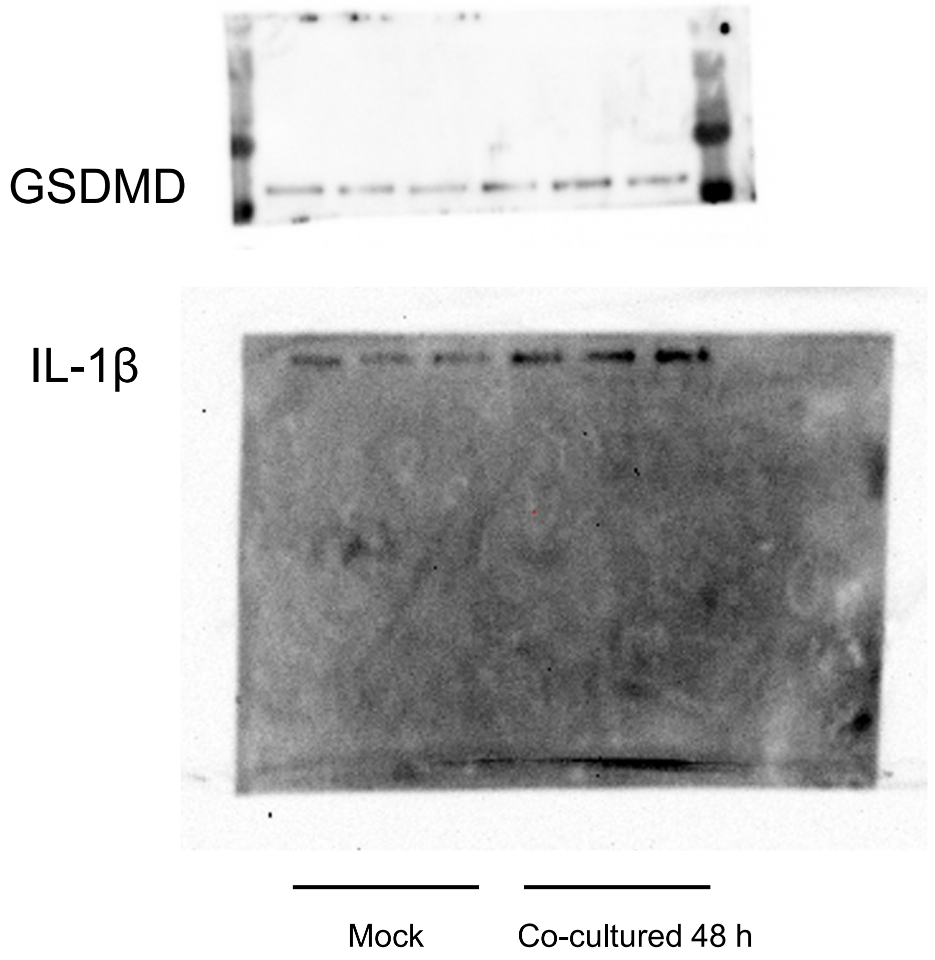

Western blotting bands in Figure 2H.

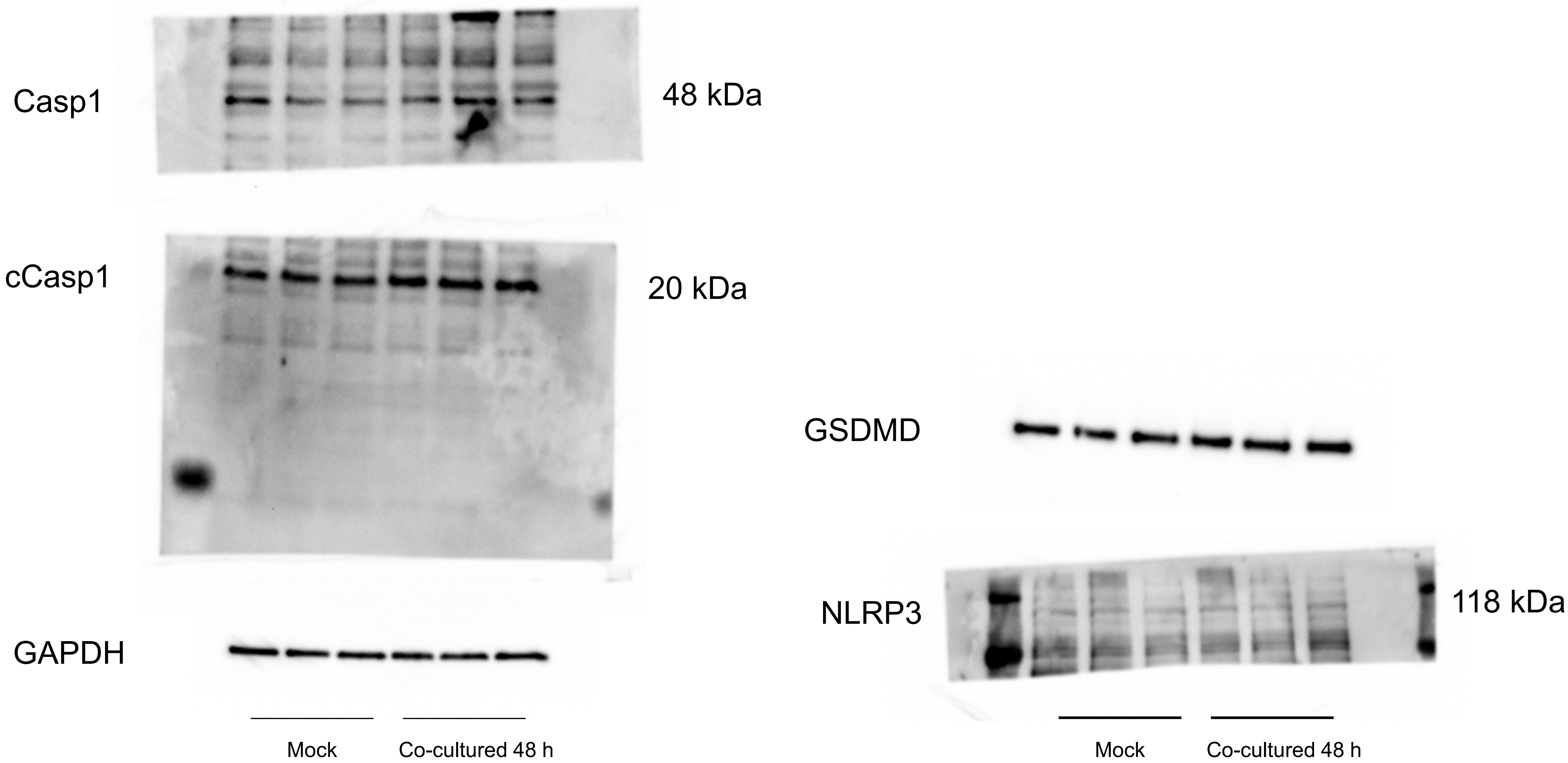

Western blotting bands in Figure 6.

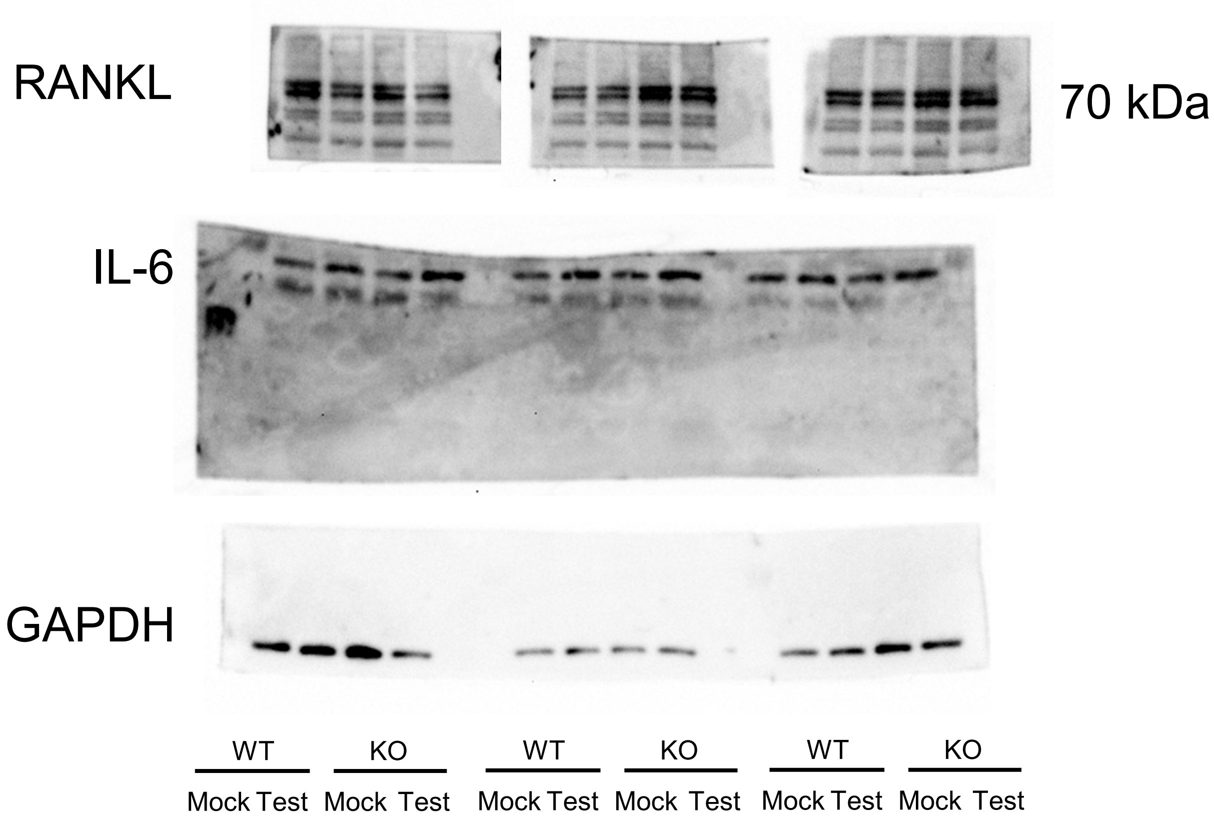

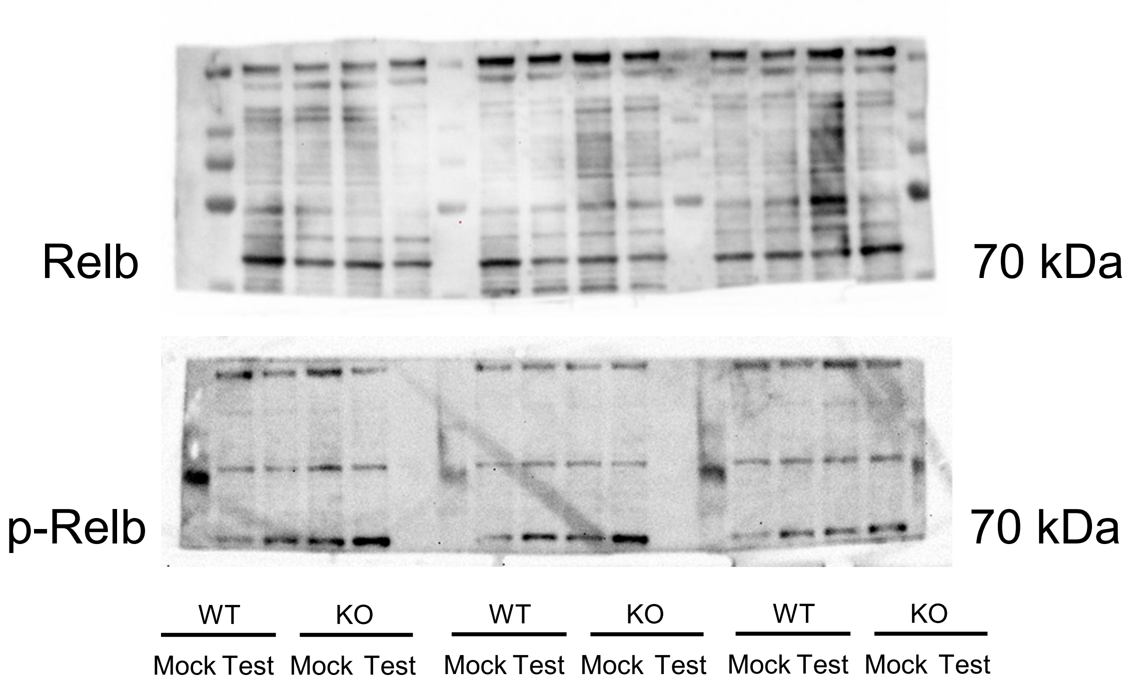

Western blotting bands in Supplementary Figure 3.

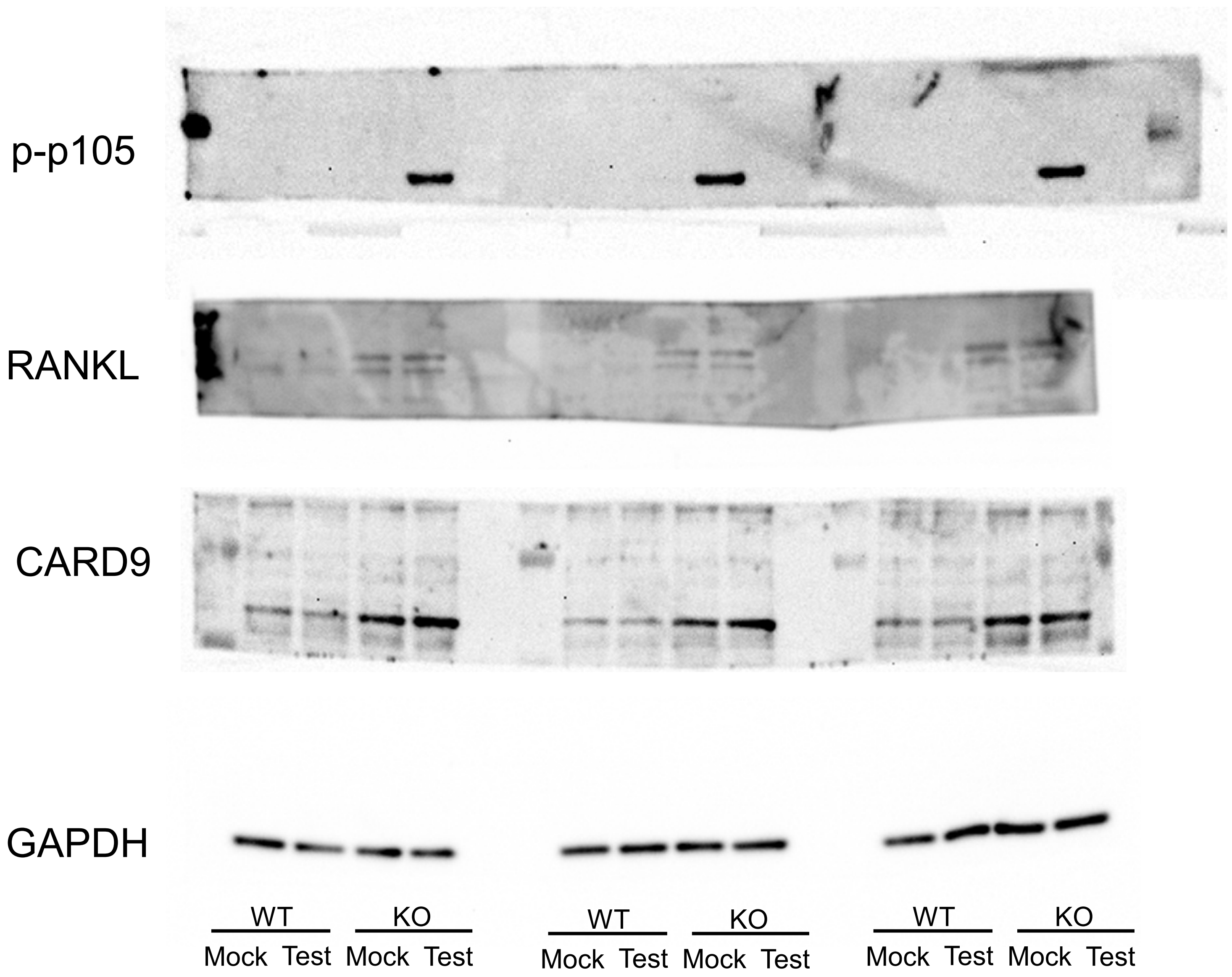

Western blotting bands in Supplementary Figure 4A.

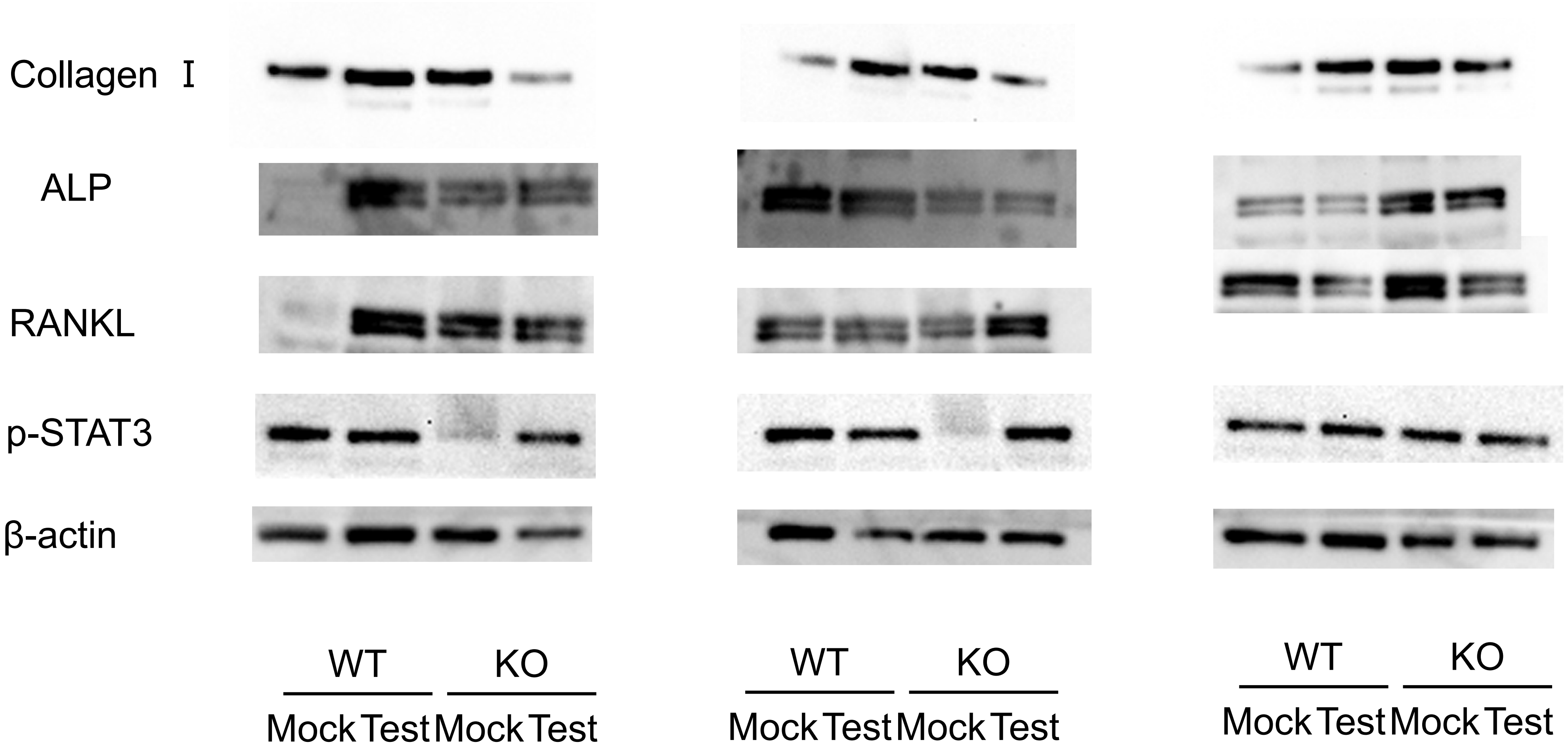

Western blotting bands in Supplementary Figure 4B.

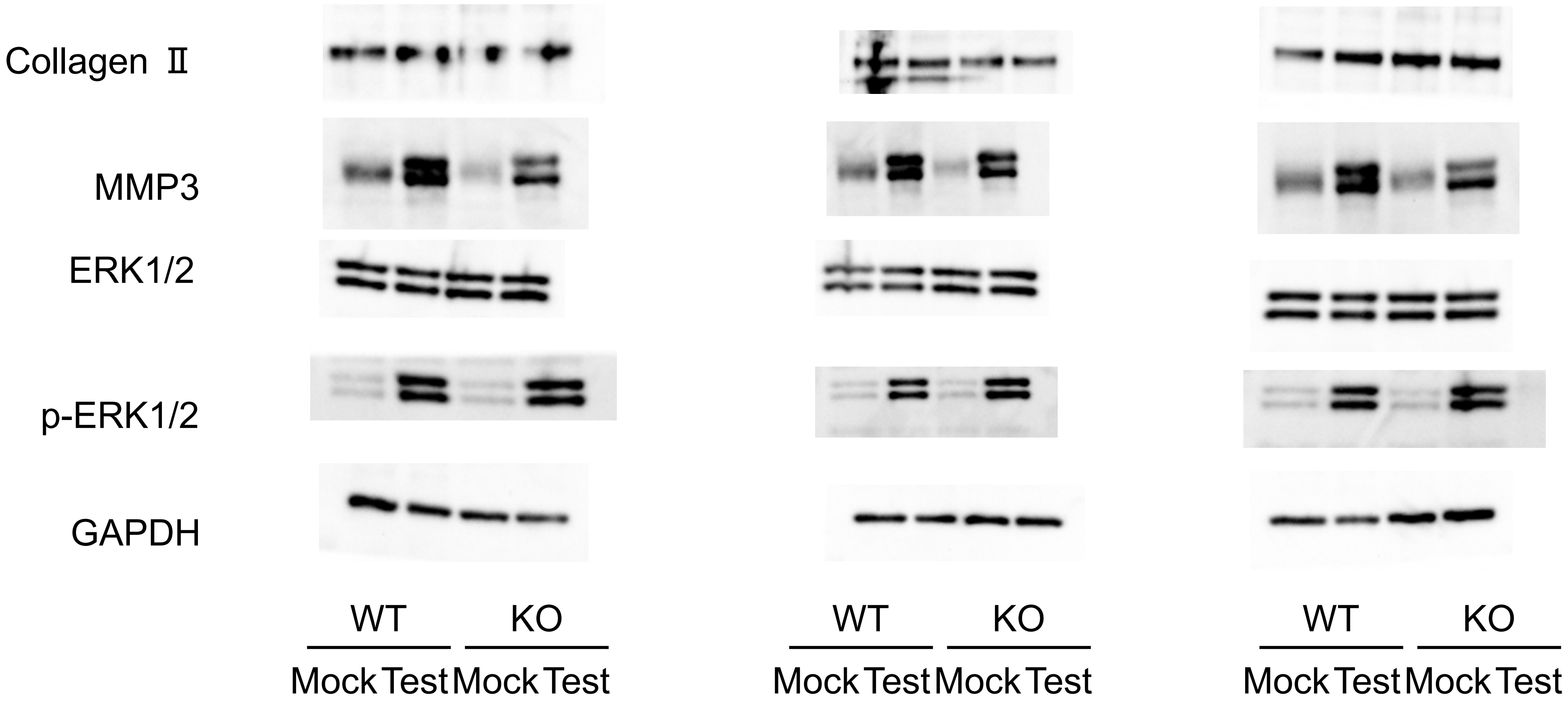

Western blotting bands in Supplementary Figure 5B.

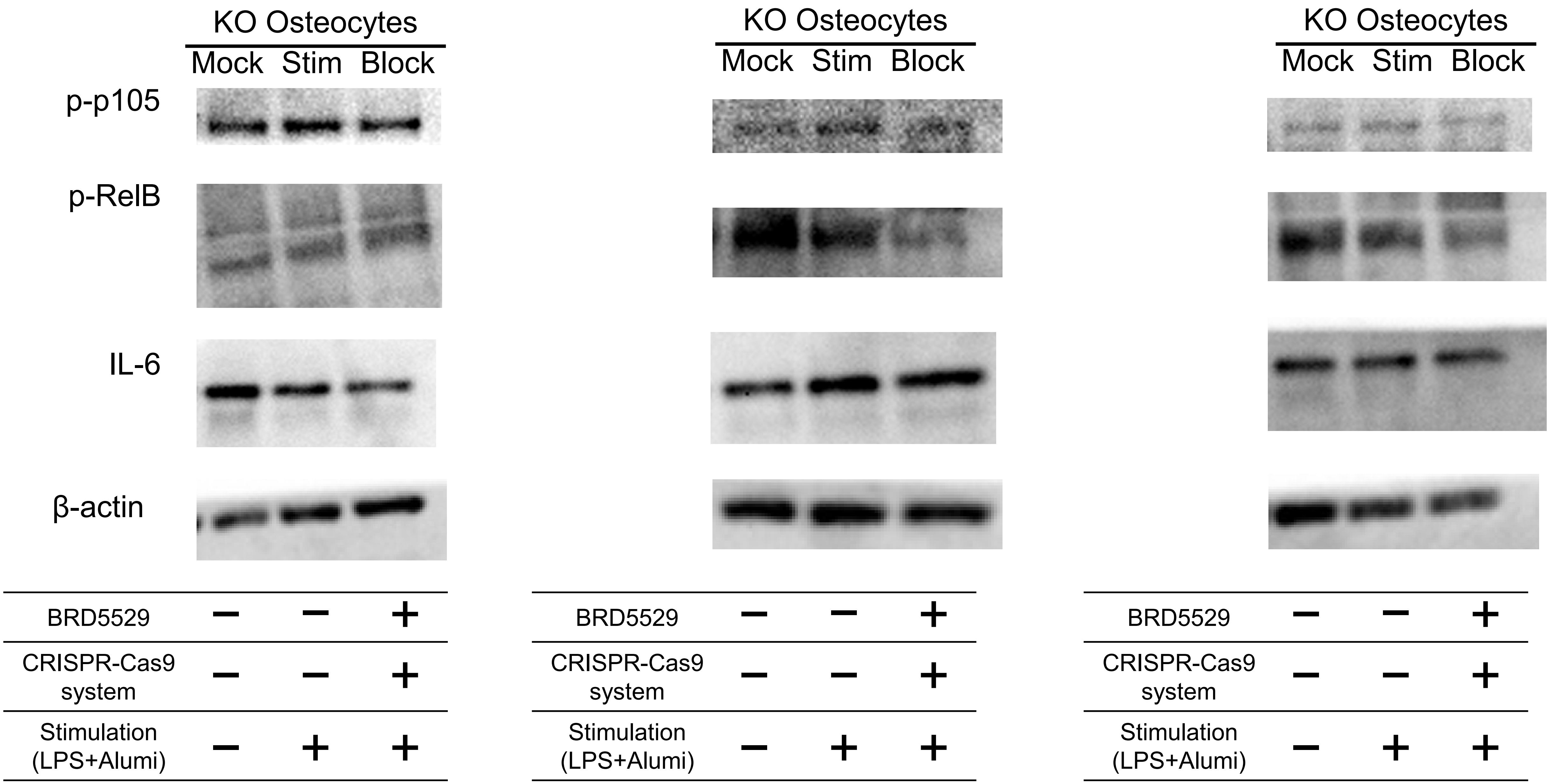

Supplement: Supplementary file 2 — Raw data [file 41413_2026_566_MOESM2_ESM.pdf]
